# Distinct tau folds initiate templated seeding and alter the post-translational modification profile

**DOI:** 10.1093/brain/awad272

**Published:** 2023-08-10

**Authors:** Airi Tarutani, Fuyuki Kametani, Marina Tahira, Yuko Saito, Mari Yoshida, Andrew C Robinson, David M A Mann, Shigeo Murayama, Taisuke Tomita, Masato Hasegawa

**Affiliations:** Department of Brain and Neurosciences, Tokyo Metropolitan Institute of Medical Science, Tokyo 156-8506, Japan; Laboratory of Neuropathology and Neuroscience, Graduate School of Pharmaceutical Sciences, The University of Tokyo, Tokyo 113-0033, Japan; Department of Brain and Neurosciences, Tokyo Metropolitan Institute of Medical Science, Tokyo 156-8506, Japan; Department of Brain and Neurosciences, Tokyo Metropolitan Institute of Medical Science, Tokyo 156-8506, Japan; Department of Neuropathology (Brain Bank for Aging Research), Tokyo Metropolitan Institute of Geratrics and Gerontology, Tokyo 173-0015, Japan; Department of Neuropathology, Institute for Medical Science of Aging, Aichi Medical University, Aichi 480-1195, Japan; Faculty of Biology, Medicine and Health, School of Biological Sciences, Division of Neuroscience, The University of Manchester, Salford Royal Hospital, Salford M6 8HD, UK; Faculty of Biology, Medicine and Health, School of Biological Sciences, Division of Neuroscience, The University of Manchester, Salford Royal Hospital, Salford M6 8HD, UK; Department of Neuropathology (Brain Bank for Aging Research), Tokyo Metropolitan Institute of Geratrics and Gerontology, Tokyo 173-0015, Japan; Brain Bank for Neurodevelopmental, Neurological and Psychiatric Disorders, United Graduate School of Child Development, Osaka University, Osaka 565-0871, Japan; Laboratory of Neuropathology and Neuroscience, Graduate School of Pharmaceutical Sciences, The University of Tokyo, Tokyo 113-0033, Japan; Department of Brain and Neurosciences, Tokyo Metropolitan Institute of Medical Science, Tokyo 156-8506, Japan

**Keywords:** tauopathy, tau, post-translational modifications, templated seeding, strains

## Abstract

Pathological tau accumulates in the brain in tauopathies such as Alzheimer's disease, Pick's disease, progressive supranuclear palsy and corticobasal degeneration, and forms amyloid-like filaments incorporating various post-translational modifications (PTMs). Cryo-electron microscopic (cryo-EM) studies have demonstrated that tau filaments extracted from tauopathy brains are characteristic of the disease and share a common fold(s) in the same disease group. Furthermore, the tau PTM profile changes during tau pathology formation and disease progression, and disease-specific PTMs are detected in and around the filament core. In addition, templated seeding has been suggested to trigger pathological tau amplification and spreading *in vitro* and *in vivo*, although the molecular mechanisms are not fully understood. Recently, we reported that the cryo-EM structures of tau protofilaments in SH-SY5Y cells seeded with patient-derived tau filaments show a core structure(s) resembling that of the original seeds. Here, we investigated PTMs of tau filaments accumulated in the seeded cells by liquid chromatography/tandem mass spectrometry and compared them with the PTMs of patient-derived tau filaments. Examination of insoluble tau extracted from SH-SY5Y cells showed that numerous phosphorylation, deamidation and oxidation sites detected in the fuzzy coat in the original seeds were well reproduced in SH-SY5Y cells. Moreover, templated tau filament formation preceded both truncation of the N-/C-terminals of tau and PTMs in and around the filament core, indicating these PTMs may predominantly be introduced after the degradation of the fuzzy coat.

## Teaser

Tarutani *et al.* compare post-translational modifications (PTMs) of tau in a cellular seeding model to those of tau filaments derived from patients with tauopathies, and show that most tau PTMs are reproduced in the model. PTMs characteristic of individual tauopathies emerge in the model as tau filaments mature.

## Introduction

Tau is a microtubule-binding protein, which functions to stabilize microtubules by promoting tubulin polymerization.^1–3^ In the adult human brain, alternative mRNA splicing of the *MAPT* gene on chromosome 17q21.31 results in the expression of six tau isoforms ranging from 352 to 441 amino acids in length.^4,5^ Tau is structurally composed of an N-terminal domain, a proline-rich domain, a microtubule-binding domain and a C-terminal domain, and is classified as three-repeat (3R) tau or four-repeat (4R) tau depending on the number of repeated sequences in the microtubule-binding domain. Neurodegenerative diseases in which pathological tau accumulates in neuronal and/or glial cells are referred to as tauopathies. The physiological function of neuronal tau is regulated by normal phosphorylation within axons, whereas pathological tau observed in tauopathy brains is abnormally phosphorylated, mislocalizes to the cell body and loses microtubule-binding ability.^6–11^ Partial amino acid sequences in the microtubule-binding and C-terminal domains of pathological tau also form a disease-specific filament core consisting of 8–13 β-strands, and these ordered assemblies appear as amyloid-like filaments.^12^ The more flexible part around the filament core is termed the fuzzy coat.^13^ Furthermore, pathological tau undergoes various post-translational modifications (PTMs), such as truncation, ubiquitination, acetylation, methylation, deamidation and oxidation as well as phosphorylation in both the filament core and the fuzzy coat.^14,15^ The PTM profile has been reported to alter concomitantly with disease progression in Alzheimer’s disease brains.^16^ Intracellular accumulation of tau filaments in tauopathy brains correlates with disease progression and expands throughout the brain along neuronal circuits.^17,18^ Prion-like propagation, in which filamentous tau self-amplifies like a prion and spreads from cell to cell, has been proposed as a mechanism underlying the stereotypical expansion of tau pathology, and has been demonstrated in both *in vitro* and *in vivo* experimental models.^19,20^ However, the molecular mechanisms of cell-to-cell transmission remain to be fully understood.

In addition, the tau isoforms that constitute tau pathology and the structural and biochemical properties of tau filaments display heterogeneity within tauopathies, suggesting the existence of pathological tau strains and associated clinicopathological diversity in tauopathies.^21^ Neurofibrillary tangles, one of the neuropathological hallmarks of Alzheimer’s disease, are formed by the neuronal accumulation of paired helical filaments (PHFs) and straight filaments (SFs) composed of 3R tau and 4R tau.^22,23^ In Pick’s disease (PiD), 3R tau accumulates in neuronal cells as Pick bodies, whereas in progressive supranuclear palsy (PSP) and corticobasal degeneration (CBD), 4R tau accumulates in neuronal and glial cells.^24,25^ Tufted astrocytes and astrocytic plaques neuropathologically define PSP and CBD, respectively.^24^ Cryo-electron microscopic (cryo-EM) studies of tau filaments extracted from tauopathy brains has enabled structural classification of tauopathies based on the atomic structure of the filament core.^12^ Tauopathies can also be biochemically classified by immunoblot analysis of sarkosyl-insoluble tau extracted from the patient’s brain.^26,27^ Intriguingly, the biochemical classification of 4R tauopathies corresponds to their structural classification. The isoform specificity and pathological features of tauopathy brains have been reported to be reproduced in seeded tau aggregation and pathology formation in *in vitro* and *in vivo* models using patient-derived tau seeds.^28–33^ Disease-defining tau filaments were also seen in multiple cases and in multiple brain regions in one individual, supporting the idea that templated amplification of tau filaments can occur in the patient’s brain.^12,34–36^ However, the involvement of PTMs in templated tau seeding and tau strain formation remains unclear.

In this study, we aimed to clarify the putative roles of disease-defining tau filament folds and PTMs in templated tau seeding. We have previously reported that patient-derived tau seeds cause strain-specific tau aggregation in SH-SY5Y cells expressing full-length tau.^30^ More recently, we showed that protofilaments that accumulate in the seeded cells present a filament fold(s) resembling that of the original seeds, suggesting that intracellular amplification of tau filaments is possibly template-dependent.^37^ Using this cellular model, the PTM profiles of insoluble tau accumulated in the cells were analysed by liquid chromatography/tandem mass spectrometry (LC-MS/MS). We show that tau PTMs in the fuzzy coat region reproduce those of the original seeds, whereas PTMs in the filament core region do not. Our results suggest that PTM events are staged in the process of tau filament/pathology formation.

## Materials and methods

### Ethics statement

Post-mortem brain tissues from patients neuropathologically diagnosed as Alzheimer’s disease, PiD, PSP and CBD were obtained from the Brain Banks in Tokyo Metropolitan Institute of Geratrics and Gerontology, the University of Manchester, National Center of Neurology and Psychiatry and Aichi Medical University. The study protocol was approved by the ethics committees of Tokyo Metropolitan Institute of Medical Science (21–1) and the University of Tokyo (30–7). All methods were performed in accordance with the relevant guidelines and regulations. All brain tissues used in this study were anonymized.

### Antibodies

Anti-tau antibodies used in this study were as follows: T46 (epitope: 404–441; Thermo Fisher Scientific), pS396 (epitope: p-Ser-396; Calbiochem), RD3 (epitope: 209–224; Millipore), Anti-4R (epitope: 275–291; Cosmobio), TauC (epitope: 429–441; Cosmobio), Tau 354–369 (epitope: 354–369; Cosmobio) and Tau 360–380 (epitope: 360–380; Cosmobio). Monoclonal anti-α-tubulin antibody (T9028) and monoclonal and polyclonal anti-HA antibodies (H3663 and H6908) were purchased from Sigma. Polyclonal anti-ubiquitin antibody (Z0458) was obtained from Dako.

### Preparation of sarkosyl-insoluble fractions from patients’ brains

For each case, a brain sample (0.5 g) was homogenized in 20 volumes (w/v) of A68 buffer (10 mM Tris-HCl pH 7.5 containing 10% sucrose, 0.8 M NaCl, 1 mM EGTA) and incubated for 30 min at 37°C, after addition of sarkosyl (final concentration: 2%). Brain homogenates were centrifuged at 27 000*g* for 10 min at 25°C, then ultracentrifuged at 113 000*g* for 20 min at 25°C. The pellets were washed with saline and ultracentrifuged as before. The resulting pellets were collected as sarkosyl-insoluble fractions of patients’ brains, resuspended in saline by sonication for 15 s and used for immunoblotting, introduction into SH-SY5Y cells and trypsin treatment. For LC-MS/MS analysis, sarkosyl-insoluble fractions were stored at −80°C until use.

### Cell culture, transfection of plasmids and introduction of pathological tau seeds into cells

Human neuroblastoma SH-SY5Y cells were maintained at 37°C in 5% CO_2_ in Dulbecco’s modified Eagle medium (DMEM)/F12 medium (Sigma-Aldrich) supplemented with 10% fetal calf serum, penicillin-streptomycin glutamine (Gibco) and MEM non-essential amino acids solution (Gibco). For the expression of ^13^C_6_-Lys-labelled tau, a SILAC Protein Quantitation Kit (LysC)-DMEM:F12 from Thermo Fisher Scientific was used. For the expression of ^13^C_6_^15^N_2_-Lys- and ^13^C_6_^15^N_4_-Arg-labelled tau, SILAC-DMEM/F12 medium and heavy amino acids were obtained from FUJIFILM and Funakoshi. Isotope labelling was performed according to the manufacturer’s instructions. Cells were cultured to 60–70% confluence in 6-well plates and transfected with plasmids using X-tremeGENE 9 (Roche Life Science) according to the manufacturer’s instructions. We used HA-tagged human tau 1N3R and 1N4R in pCDNA3.1 vector. After transfection of plasmids, cells were incubated for 8 h, and 1–2 μl of the diluted sarkosyl-insoluble fractions extracted from patients’ brains or seeded SH-SY5Y cells was introduced using MultiFectam (Promega) according to the manufacturer’s instructions. Transfected cells were incubated for 1, 3 or 5 days.

### Preparation of sarkosyl-insoluble fractions from SH-SY5Y cells

Transfected SH-SY5Y cells were washed with PBS, and extracted with 1 ml of 1% sarkosyl in A68 buffer. Cell extracts were sonicated for 15 s. After incubation for 30 min at 37°C, cell extracts were ultracentrifuged at 113 000*g* for 20 min at 25°C. The supernatants were removed and collected as sarkosyl-soluble fractions, then the pellets were washed with 30 mM Tris-HCl (pH 7.5) and ultracentrifuged as before. The resulting pellets were collected as sarkosyl-insoluble fractions. For immunoblotting, immunoelectron microscopy, trypsin treatment and secondary seeded aggregation, sarkosyl-insoluble fractions were resuspended in 30 mM Tris-HCl (pH 7.5) and sonicated for 15 s. For LC-MS/MS analysis, the sarkosyl-insoluble fractions were stored at −80°C until use.

### Trypsin treatment of sarkosyl-insoluble fractions

Sarkosyl-insoluble fractions extracted from patients’ brains and SH-SY5Y cells were treated with trypsin at a final concentration of 0.1–1 μg/μl at 37°C for 30 min, and the trypsinized samples were used for immunoblotting. For trypsinized patient-derived tau seeds, trypsin was added at a final concentration of 0.1 μg/μl After incubation at 37°C for 0, 15 or 60 min, the digestion was stopping by boiling. Trypsinized samples were used for immunoblotting. The sarkosyl-insoluble fractions remaining after trypsinization for 60 min, were used as trypsinized seeds for introduction into SH-SY5Y cells.

### Immunoblotting

Immunoblotting was performed as described.^27^ Sarkosyl-insoluble and -soluble fractions and trypsinized samples were added to SDS-sample buffer and boiled for 3 min. The samples were separated on 4–20% gradient polyacrylamide gel (Wako) and 10% or 15% polyacrylamide gels. The protein concentrations of samples extracted from SH-SY5Y cells were determined with a BCA Protein Assay Kit (Thermo Fisher Scientific). Monoclonal anti-α-tubulin was used to obtain a loading control. The band intensities of immunoblots were quantified using ImageQuant TL (Cytiva) and were analysed using Prism software (GraphPad Software).

### Immunoelectron microscopy

Immunoelectron microscopy was performed as described.^38^ Briefly, sarkosyl-insoluble fractions extracted from the seeded cells were dropped onto carbon-coated 300-mesh copper grids (Nissin EM) and dried. The grids were immunostained with anti-HA antibody (1:100) and secondary antibody conjugated to 5 nm gold particles (Cytodiagnostics, 1:50). The immunostained grids were negatively stained with a drop of 2% phosphotungstic acid and dried. Electron micrograph images were recorded with a JEOL JEM-1400 electron microscope.

### LC-MS/MS analysis of sarkosyl-insoluble fractions extracted from patients’ brains and SH-SY5Y cells

Sarkosyl-insoluble fractions were treated with 99% formic acid for 1 h at room temperature, then diluted in ultrapure water and dried in a Speed-Vac (Savant). Sarkosyl in the sarkosyl-soluble fractions was removed by using Pierce^®^ Detergent Removal Spin Columns (Thermo Fisher Scientific). The denatured sarkosyl-insoluble fraction and the detergent-free sarkosyl-soluble fraction were incubated in 50 μM triethylammonium bicarbonate buffer (Fluka) containing 4 μg trypsin/Lys-C mix (Promega) at 37°C for 20 h. The trypsinized samples were boiled for 5 min after addition of DTT (final concentration: 3 μM) and dried in a Speed-Vac. Peptides were resuspended in 0.1% formic acid and analysed by LC-MS/MS as described.^15^ The data were analysed with Proteome Discoverer (Thermo Fisher Scientific Inc., Waltham, USA), Mascot software (Matrix Science Inc., Boston, USA) and Scaffold software (Proteome Software, Inc., Oregon, USA). The sequences were extracted from Swissprot and GenBank databases.

## Results

### Tau filament formation in SH-SY5Y cells seeded with patient-derived tau strains

**Figure 1:**
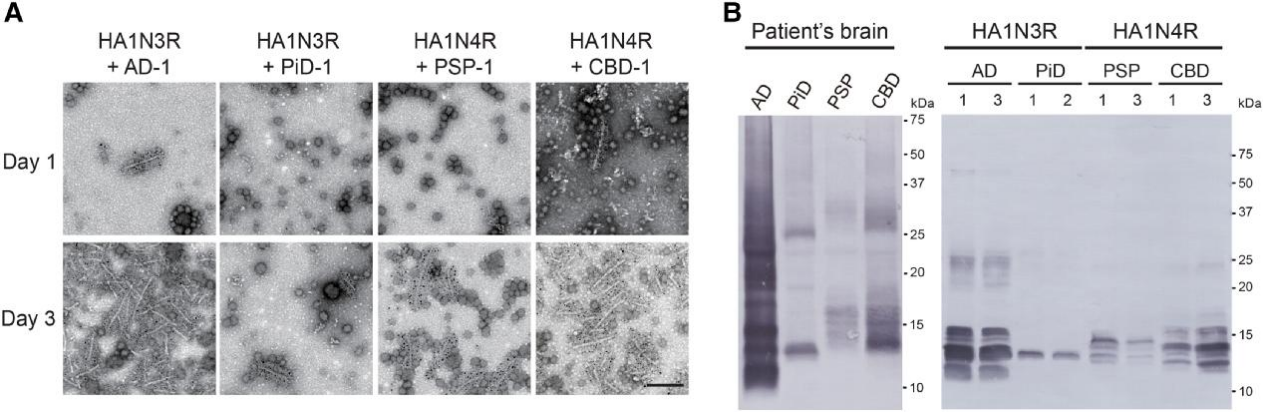
**Seeded tau filament formation in SH-SY5Y cells**. (**A**) Immunoelectron microscopy of sarkosyl-insoluble fractions extracted from SH-SY5Y cells on Days 1 and 3 after seeding with patient-derived tau seeds. Electron micrographs show fibrous structures positive for anti-HA antibody, that were labelled with the secondary antibody conjugated to 5 nm gold particles. Scale bar = 200 nm. (**B**) Trypsin-resistant tau banding patterns of sarkosyl-insoluble fractions extracted from tauopathy brains and SH-SY5Y cells seeded with patient-derived tau seeds are shown. Trypsin-resistant tau bands were detected by immunoblot analysis with Tau 360–380 antibody.

First, we investigated whether tau filaments amplified in the cellular seeding model maintained the structural characteristics of patient-derived tau strains. Sarkosyl-insoluble fractions were extracted from cases neuropathologically diagnosed as Alzheimer’s disease (AD), PiD, PSP or CBD (AD-tau, PiD-tau, PSP-tau, CBD-tau) and introduced into SH-SY5Y cells transiently expressing HA-tagged 3R tau with 1N insert (HA1N3R) or HA-tagged 4R tau with 1N insert (HA1N4R) (Table 1 and Supplementary Fig. 1). Numerous filament structures labelled with HA antibody were observed in the sarkosyl-insoluble fraction extracted from the cells at 3 days after introduction, whereas few filaments were seen at one day (Fig. 1A). Furthermore, trypsin treatment of the insoluble fraction extracted from the seeded cells was performed and the trypsin-resistant tau banding pattern was examined. Immunoblot analysis with tau 360–380 antibody recognizing the filament core region revealed various sizes of trypsin-resistant tau, 16, 15, 13 and 12 kDa in seeded AD-tau, 13 kDa in seeded PiD-tau, 14, 13.5, 13, and 12.5 kDa in seeded PSP-tau and 16, 13.5, 13 and 12.5 kDa in seeded CBD-tau (Fig. 1B and Supplementary Fig. 2). The production of distinct trypsin-resistant tau depending on the seeds used suggests that the tau filaments formed in SH-SY5Y cells are conformationally different. These trypsin-resistant tau banding patterns also resembled those of the patient-derived insoluble tau (Fig. 1B).^27^ These results, together with the structural similarities to patient-derived tau filaments revealed by cryo-EM analysis,^37^ indicate that tau filament formation in SH-SY5Y cells occurs in a template-dependent manner.

**Table 1:** Neuropathological information for tauopathy brains used in this study

Diagnosis	Case number	Gender	Age at death (years)	Duration (years)	Brain (g)	PMI (h)
AD	1	Female	65	9	1165	N/A
AD	2	Female	94	15	983	11
AD	3	Male	56	4	1358	N/A
AD	4	Male	85	N/A	1146	8
AD	5	Female	59	N/A	985	3
AD	6	Female	85	10	1120	35.5
AD	7	Male	74	13	1140	19
PiD	1	Female	62	10	928	N/A
PiD	2	Male	56	10	1150	N/A
PiD	3	Female	58	8	1065	26
PiD	4	Female	80	10	1080	N/A
PiD	5	N/A	N/A	N/A	N/A	N/A
PSP	1	Male	78	18	N/A	19
PSP	2	Female	88	12	N/A	24
PSP	3	Male	82	N/A	1280	3
PSP	4	Male	75	3.5	1080	5
PSP	5	Male	74	8	1334	47
PSP	6	Female	86	11	1250	2.5
CBD	1	Female	74	6	899	N/A
CBD	2	Male	73	N/A	1200	3
CBD	3	Female	74	35	N/A	N/A
CBD	4	Male	75	3.3	1041	2.5
CBD	5	Male	79	6	1266	12.5
CBD	6	Female	75	12	936	5

AD = Alzheimer’s disease; CBD = corticobasal degeneration; PiD = Pick’s disease; PMI = post-mortem interval; PSP = progressive supranuclear palsy.

### PTMs of tau filaments in SH-SY5Y cells seeded with patient-derived tau strains

**Figure 2:**
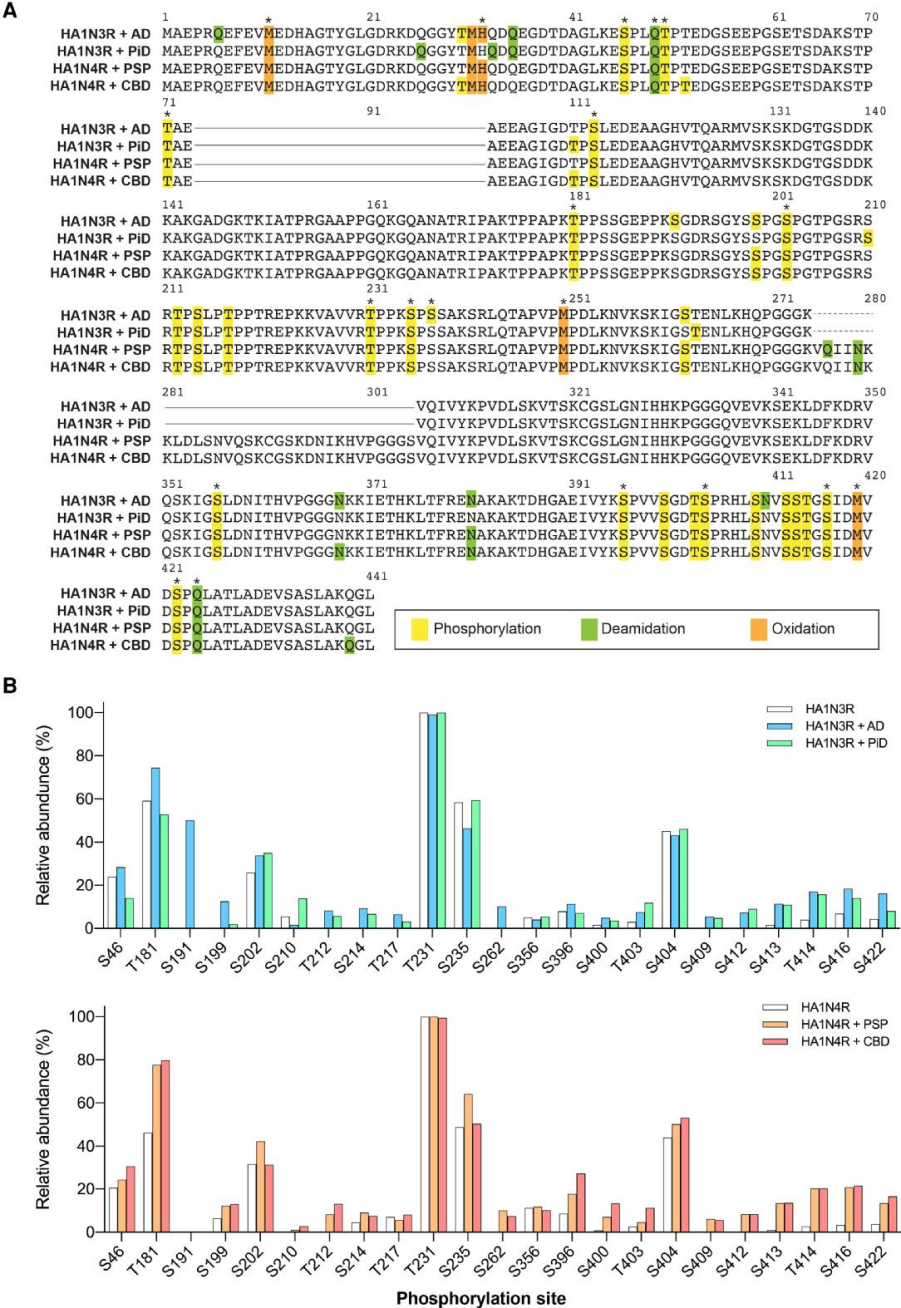
**Tau post-translational modifications (PTMs) of insoluble tau extracted from SH-SY5Y cells seeded with patient-derived tau strains.** (**A**) Sequence alignments of 1N3R in sarkosyl-insoluble tau extracted from SH-SY5Y cells expressing HA1N3R seeded with AD-tau (HA1N3R + AD) and PiD-tau (HA1N3R + PiD), and 1N4R in sarkosyl-insoluble tau extracted from SH-SY5Y cells expressing HA1N4R seeded with PSP-tau (HA1N4R + PSP) and CBD-tau (HA1N4R + CBD). PTMs detected by liquid chromatography-tandem mass spectrometry at >3% relative abundance are shown. Asterisks indicate PTM sites detected at >3% relative abundance in both sarkosyl-insoluble tau extracted from SH-SY5Y cells expressing HA1N3R and cells expressing HA1N4R shown in Supplementary Fig. 4A. (**B**) Relative abundances of major tau phosphorylation sites detected in the sarkosyl-insoluble fractions extracted from SH-SY5Y cells expressing HA1N3R without seeds (HA1N3R) and seeded with AD-tau (HA1N3R + AD) and PiD-tau (HA1N3R + PiD) (*top*) and from SH-SY5Y cells expressing HA1N4R without seeds (HA1N4R) and seeded with PSP-tau (HA1N4R + PSP) and CBD-tau (HA1N4R + CBD) (*bottom*). Relative abundance (%) was calculated from the ratio of modified peptides/(modified + unmodified peptides). The results are expressed as means (*n* = 3–5). AD = Alzheimer’s disease; CBD = corticobasal degeneration; PiD = Pick’s disease; PSP = progressive supranuclear palsy.

Consistent with the presence of structural differences in tau filaments, disease-specific PTMs in and around the filament core are found in insoluble tau extracted from tauopathy brains.^15^ Therefore, to characterize the PTM profile associated with templated tau seeding, PTMs of insoluble tau extracted from SH-SY5Y cells were investigated. The sarkosyl-insoluble fractions were denatured with formic acid and digested with trypsin, and the resulting peptides were analysed by LC-MS/MS. To distinguish between tau derived from the introduced seeds and tau expressed in the cells, we employed the stable isotope labelling using amino acids in cell culture (SILAC) system and introduced patient-derived tau seeds into SH-SY5Y cells expressing isotopically labelled HA1N3R or HA1N4R (heavy-HA1N3R or heavy-HA1N4R). Only small numbers of tau peptides, probably derived from the introduced seeds, were detected in the sarkosyl-insoluble fractions extracted from seeded SH-SY5Y cells not expressing tau (Supplementary Fig. 3A and Supplementary Table 1). In contrast, numerous isotopically labelled tau peptides were obtained from the sarkosyl-insoluble fractions extracted from SH-SY5Y cells expressing heavy tau without and with the introduction of patient-derived tau seeds (Supplementary Fig. 3B). The relative abundance of each PTM was calculated from the percentage of modified peptides in all peptides obtained from one sample, and only PTMs with a relative abundance of >3% are presented below, unless otherwise stated. We observed phosphorylation at 27 sites (T30, S46, T50, T71, S113, T181, S191, S199, S202, T212, S214, T217, T231, S235, S237, S262, S356, S396, S400, T403, S404, S409, S412, S413, T414, S416 and S422) in insoluble tau extracted from SH-SY5Y cells expressing heavy-HA1N3R at 3 days after introduction of AD-tau in four cases (HA1N3R-AD) (Fig. 2, Supplementary Fig. 4B and Supplementary Table 2). Furthermore, phosphorylation at 25 sites (S46, T50, T71, T111, S113, T181, S202, S210, T212, S214, T217, T231, S235, T263, S356, S396, S400, T403, S404, S409, S412, S413, T414, S416 and S422) was detected in insoluble tau extracted from SH-SY5Y cells expressing heavy-HA1N3R after introduction of PiD-tau in four cases (HA1N3R-PiD) (Fig. 2, Supplementary Fig. 4B and Supplementary Table 3). Insoluble tau extracted from SH-SY5Y cells only expressing heavy-HA1N3R (HA1N3R) showed 18 phosphorylation sites (S46, T50, T69, T71, S113, T181, S202, T231, S235, S237, S356, S396, S400, T403, S404, T414, S416 and S422) (Fig. 2B, Supplementary Fig. 4 and Supplementary Table 4). Similarly, phosphorylation at 24 sites (S46, T50, T71, S113, T181, S199, S202, T212, S214, T217, T231, S235, S262, S356, S396, S400, T403, S404, S409, S412, S413, T414, S416 and S422) was detected in insoluble tau extracted from SH-SY5Y cells expressing heavy-HA1N4R at 3 days after introduction of PSP-tau in four cases (HA1N4R-PSP) (Fig. 2, Supplementary Fig. 4B and Supplementary Table 5). Phosphorylation at 27 sites (T30, S46, T50, T52, T71, T111, S113, T181, S199, S202, T212, S214, T217, T231, S235, S262, S356, S396, S400, T403, S404, S409, S412, S413, T414, S416 and S422) was detected in insoluble tau extracted from SH-SY5Y cells expressing heavy-HA1N4R after introduction of CBD-tau in four cases (HA1N4R-CBD) (Fig. 2, Supplementary Fig. 4B and Supplementary Table 6). There were 20 phosphorylation sites (T30, T39, S46, T50, T71, S113, T181, S199, S202, S214, T217, T231, S235, S237, T263, S356, S396, S404, S416 and S422) in insoluble tau extracted from SH-SY5Y cells only expressing heavy-HA1N4R (HA1N4R) (Fig. 2B, Supplementary Fig. 4 and Supplementary Table 7). Tau PTMs detected in the sarkosyl-soluble and sarkosyl-insoluble fractions extracted from cells only expressing heavy-HA1N3R or heavy-HA1N4R did not show remarkable differences (Supplementary Tables 4, 7 and 8). In summary, phosphorylation at T30, T52, T111, S191, S199, S210, T212, S214, T217, S262, T263, S400, T403, S409, S412, S413 and T414 was detected apparently in insoluble tau from cells in which filamentous tau was accumulated. The relative abundance of phosphorylation at T50, T181, S396, S416 and S422 was also increased after seeded tau aggregation. These results indicate that tau expressed in SH-SY5Y cells is highly phosphorylated, and that the phosphorylation sites and frequencies detected in the proline-rich domain, microtubule-binding domain and C-terminal domain increase concomitantly with intracellular tau filament formation and accumulation.

Deamidation was detected at seven sites (Q6, Q35, Q49, N368, N381, N410 and Q424) in HA1N3R-AD and five sites (Q26, Q33, Q35, Q49 and Q424) in HA1N3R-PiD, while deamidation at Q33, Q49 and Q424 was detected in HA1N3R (Fig. 2A, Supplementary Fig. 4 and Supplementary Tables 2–4). Similarly, five deamidation sites at Q49, Q276, Q279, N381 and Q424 in HA1N4R-PSP and six sites at Q49, N279, N368, N381, Q424 and Q439 in HA1N4R-CBD were detected, among which deamidation at Q49, N368 and Q424 was detected in HA1N4R (Fig. 2A, Supplementary Fig. 4 and Supplementary Tables 5–7). These results show that intracellular accumulation of tau filaments increases deamidation in the microtubule-binding and C-terminal domains. Oxidation at M11, M31, H32, M250 and M419 was detected in the cells both with and without filamentous tau accumulation, with comparable frequencies (Fig. 2A, Supplementary Fig. 4 and Supplementary Tables 2–7).

We have previously reported that trypsin-treated patient-derived tau seeds, from which the fuzzy coat had been removed, cause seeded tau aggregation as effectively as untreated seeds.^30^ Therefore, PTMs in insoluble tau extracted from SH-SY5Y cells seeded with trypsin-treated tau seeds were also examined (Supplementary Fig. 5 and Supplementary Table 9). The PTM profiles did not differ significantly from those in insoluble tau from cells seeded with untreated seeds (Supplementary Table 9). These results support the idea that the detected PTMs are associated with seeded aggregation of full-length tau induced by the core part of patient-derived tau filaments.

Although insoluble tau extracted from seeded SH-SY5Y cells formed filaments (Fig. 1A), PTMs such as ubiquitination and acetylation at lysine residues in the filament core region, which are prominent in patient-derived tau filaments, were below the detection limit (Fig. 2A and Supplementary Tables 2, 3, 5 and 6). All ubiquitin peptides detected in the sarkosyl-insoluble fraction extracted from SH-SY5Y cells were not ubiquitinated, in contrast to the polyubiquitination of similar peptides from tauopathy brains (data not shown).

### Comparison of PTMs in tau filaments extracted from tauopathy brains and SH-SY5Y cells

**Figure 3:**
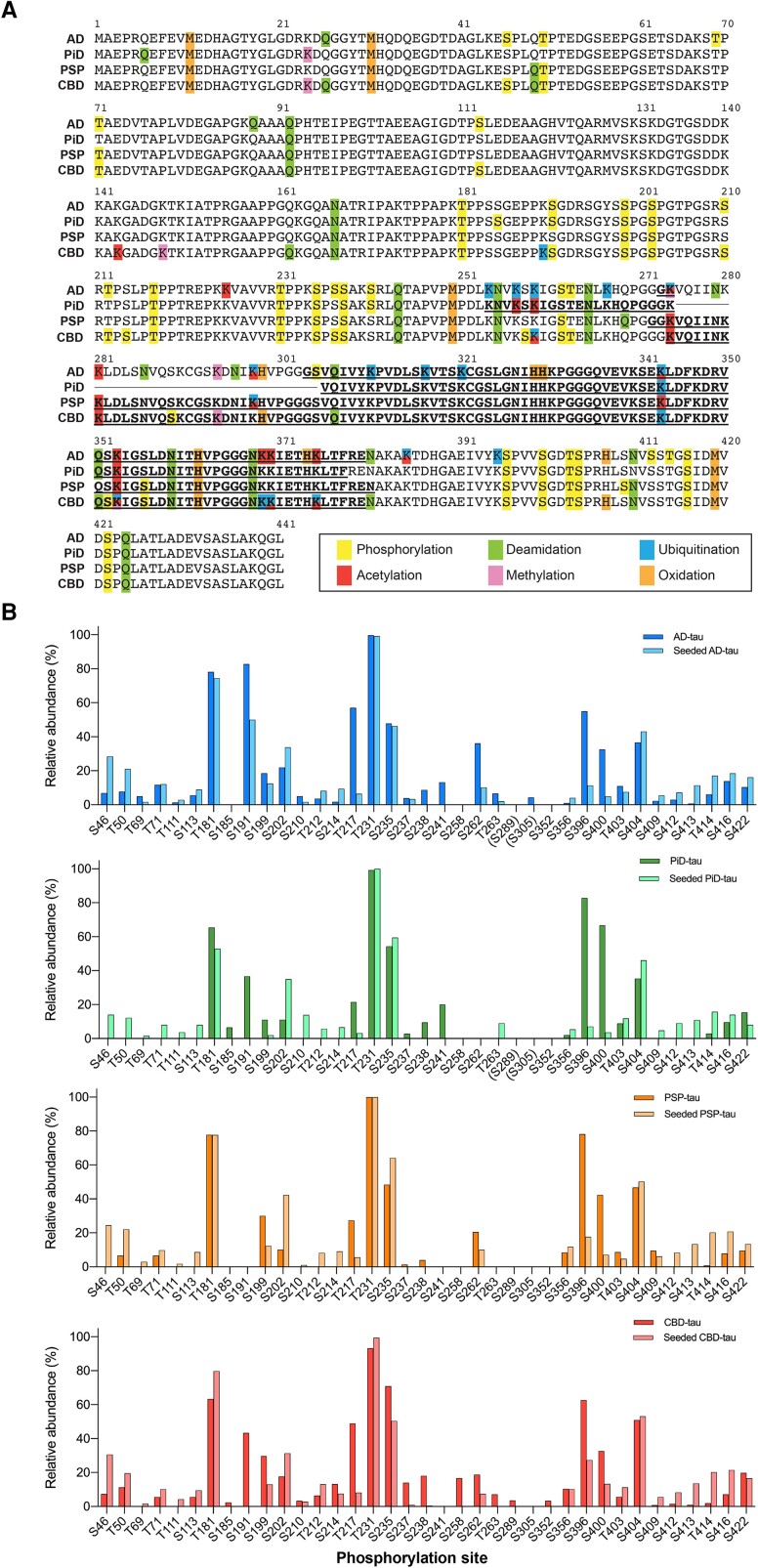
**Post-translational modifications of insoluble tau extracted from tauopathy brains**. (**A**) Sequence alignments of 2N4R in sarkosyl-insoluble tau extracted from the brains of patients with Alzheimer’s disease (AD); Pick’s disease (PiD); progressive supranuclear palsy (PSP) and corticobasal degeneration (CBD). Post-translational modifications detected by liquid chromatography-tandem mass spectrometry at >3% relative abundance are shown. Bold and underlined residues indicate the filament core regions. (**B**) Comparison of relative abundances of phosphorylation sides detected in sarkosyl-insoluble tau derived from tauopathy brains and from SH-SY5Y cells seeded with patient-derived tau (seeded tau). Relative abundance (%) was calculated from the ratio of modified peptides/(modified + unmodified peptides). The results are expressed as means (*n* = 4–6). Residues not included in seeded tau are shown in parentheses.

Furthermore, PTMs in seeded tau derived from SH-SY5Y cells were compared with PTMs of the original seeds. We examined tau PTMs in the sarkosyl-insoluble fractions extracted from five to six cases each of Alzheimer’s disease, PiD, PSP and CBD brains in the same way as described for those from SH-SY5Y cells (Fig. 3A, Table 1, Supplementary Fig. 1 and Supplementary Tables 10–13). PTMs, including phosphorylation, deamidation, oxidation, ubiquitination, acetylation and methylation, detected in patient-derived insoluble tau were consistent with those previously reported in tauopathy brains (Fig. 3A and Supplementary Tables 10–13).^15^ As previously reported, disease-specific PTMs, ubiquitination at K311, K317 and K321 and acetylation at K375 in the Alzheimer’s disease fold (G273/G304-E380 in 3R/4R tau), non-phosphorylation at S262 in the PiD fold (K254-F378 in 3R tau), less ubiquitination in the PSP fold (G272-N381 in 4R tau) and ubiquitination at K353, K369, K370 and K375 in the CBD fold (K274-E380 in 4R tau) were also identified (Fig. 3A and Supplementary Tables 10–13).^15^ Lysine PTM sites are located outside the structured filament core, except for ubiquitination/acetylation at K298 in PSP-tau, and acetylation at K281, methylation at K294 and ubiquitination/acetylation at K370 in CBD-tau (Supplementary Fig. 6). Most phosphorylation sites detected in the fuzzy coat of patient-derived insoluble tau were also present in seeded insoluble tau (Figs 2A and 3 and Supplementary Tables 2, 3, 5, 6 and 10–13). However, phosphorylation in the proline-rich and microtubule-binding domains, including S185, S191, S238, S241, S258, S289 and S352, was either undetectable or detected only at a low frequency in the seeded tau (Fig. 3B). On the other hand, phosphorylation at most sites in the N-terminal region including S46, T50, T69, T71, T111 and S113 and the C-terminal region after S409, including S409, S412, S413, S414 and S416, was detected at a higher frequency in seeded tau than in patient-derived tau (Fig. 3B). The relative abundance of phosphorylation at T217, S396 and S400 was significantly higher in patient-derived tau (Fig. 3B). Phosphorylation at T30, T52 and T111 was detectable only in seeded tau, but not in the patient-derived tau (Figs 2A and 3B). Phosphorylation at S262 was absent in both patient-derived PiD-tau and seeded PiD-tau (Fig. 3B). Deamidation and oxidation sites detected only in patient-derived insoluble tau were focused in and around the filament core. Deamidation at Q162, N167, Q244, N255, N265, Q269, N286, N296, N307 and Q351 and oxidation at H299, H329, H330, H362, H374 and H407 were detected only in the patient-derived tau (Supplementary Fig. 7). These results indicate that the degree of fuzzy coat processing differs between seeded tau and patient-derived tau, and that PTMs in and around the filament core region may predominantly be introduced after the degradation of the fuzzy coat.

### PTMs of tau identified in the seeded SH-SY5Y cells after 5 days incubation

Next, to investigate whether the increased incubation time after the addition of seeds extends the PTM profile, SH-SY5Y cells seeded with AD-tau and CBD-tau were incubated for 5 days and the PTMs of the seeded tau were examined (Supplementary Fig. 8 and Supplementary Tables 14 and 15). Additional incubation increased the relative abundance of phosphorylation at T50, S199, T212, T217, S262, S396, T403, S404 and S416 (Supplementary Fig. 8C and Supplementary Tables 14 and 15). Phosphorylation at Y29, T39, T52, T69, T175, S185, S191, Y197, S198, T205, S210, S238, S400 and S422 in seeded AD-tau and at T63, S185, S262 T263, S412 and S413 in seeded CBD-tau, and deamidation at Q33, N265, N359, N368 and Q439 in seeded AD-tau, which were not detected at 3 days after seeding with the same cases, were also detected. While the longer incubation time promoted phosphorylation and deamidation, PTMs at lysine residues remained below the detection limit (Supplementary Fig. 8C and Supplementary Tables 14 and 15).

### PTMs of tau identified in SH-SY5Y cells after secondary seeded aggregation

Lastly, we examined whether secondary seeded aggregation would make the PTM profile more representative of that of the original seeds, sarkosyl-insoluble fractions extracted from SH-SY5Y cells seeded with AD-tau, PSP-tau and CBD-tau were introduced as secondary seeds into the SH-SY5Y cells (Supplementary Fig. 9A). In addition to phosphorylation, oxidation and deamidation detected after the initial seeding (first seeding), ubiquitination and methylation were detected in insoluble tau obtained after the secondary seeding (second seeding) (Supplementary Fig. 9B and Supplementary Table 16). Consistent with the detection of ubiquitination, immunoblot analysis with anti-ubiquitin antibody showed that more ubiquitinated proteins were detected in the sarkosyl-insoluble fractions after the second seeding (Supplementary Fig. 9A). Ubiquitination at K257 in secondary-seeded AD-tau (second-seeded AD-tau), at K257, K267 and K353 in secondary-seeded PSP-tau (second-seeded PSP-tau) and at K257, K259 and K267 in secondary-seeded CBD-tau (second-seeded CBD-tau) was detected (Supplementary Fig. 9B and Supplementary Table 16). Methylation at K24 in second-seeded PSP-tau and at K44 in second-seeded CBD-tau was also detected (Supplementary Fig. 9B and Supplementary Table 16). These ubiquitinated and methylated residues are located in the fuzzy coat, except for K353. Ubiquitination at K395 in second-seeded PSP-tau and at K240, K254 and K395 in second-seeded CBD-tau, and methylation at K44 in second-seeded PSP-tau and at K369 in second-seeded CBD-tau were detected at a relative abundance of <3% (Supplementary Fig. 9B and Supplementary Table 16). Also, compared to the situation after the first seeding, phosphorylation at S46, T50, T52, S56, T175, S185, S191, S195, Y197, S198, T205, S208, S210, S212, S214, T217, S237, S396, S400, S404, S412 and S416 and deamidation at Q35, N265, N279, N286, N359, N368 and Q424 were increased after the second seeding (Supplementary Fig. 9B and Supplementary Table 16). These results indicate that after templated tau filament formation in the cells, the sites and types of PTMs tend to increase around the filament core region and more closely mimic those of the original seeds.

## Discussion

In this study, tau PTMs associated with templated seeding in SH-SY5Y cells expressing full- length tau were investigated and compared to those of the original seeds. Tau PTMs, including phosphorylation, deamidation and oxidation, in the fuzzy coat of patient-derived tau seeds was closely reproduced in seeded tau, whereas PTMs in the filament core region, especially ubiquitination and acetylation, were poorly detected (Figs 2A and 3A). Our results show that tau filaments extracted from tauopathy brains trigger templated tau filament formation and associated changes in tau PTM profiles in cultured cells.

The phosphorylation detected in tauopathy brains was well recapitulated in the seeded SH-SY5Y cells (Fig. 3B), which is consistent with the idea that an increase in phosphorylation is one of the PTM events that occurs in the early stages of tauopathy.^16,39,40^ Most of the phosphorylation, including pT181, pS202 and pT231, is also detected in SH-SY5Y cells expressing full-length tau, suggesting that templated tau seeding in this cellular model is initiated by the presence of the template rather than by phosphorylation (Supplementary Fig. 4A). Such tau phosphorylation without pathology formation has also been identified in Sf9 cells expressing 2N4R, in wild-type mouse and in human amyloid precursor protein transgenic mouse brains, as well as in healthy human brains.^16,41,42^ Furthermore, most phosphorylation, deamidation and oxidation sites that are detected only in the patient’s brain are found in and around the filament core region, indicating that seeded tau filament formation likely precedes these PTMs in SH-SY5Y cells (Fig. 3B and Supplementary Fig. 7). The lack of phosphorylation at S262 in PiD brains is explained by the location of S262 in the PiD fold, where it is inaccessible from the outside, and this was reproduced in seeded PiD-tau (Fig. 3B).^35^ Although lysine PTMs were not detected after the first seeding, detection of ubiquitination and methylation in the fuzzy coat of second-seeded tau suggests that lysine PTMs occur from the fuzzy coat towards the filament core, possibly reflecting accessibility from the outside (Supplementary Fig. 9B). An increase in phosphorylation and deamidation in and around the filament core region associated with filament formation was also observed between the first and second seedings in SH-SY5Y cells (Fig. 2B and Supplementary Fig. 9B). Given that most PTMs at lysine residues in patient-derived tau folds are detected outside the structured filament core, PTMs in the filament core region probably appear after templated seeding and are dependent on the core structure (Supplementary Fig. 6). Deamidation at N279 in seeded CBD-tau, which is not detected in CBD brains, is consistent with structural differences between CBD and seeded CBD filaments (Supplementary Fig. 7).^37,43^

**Figure 4:**
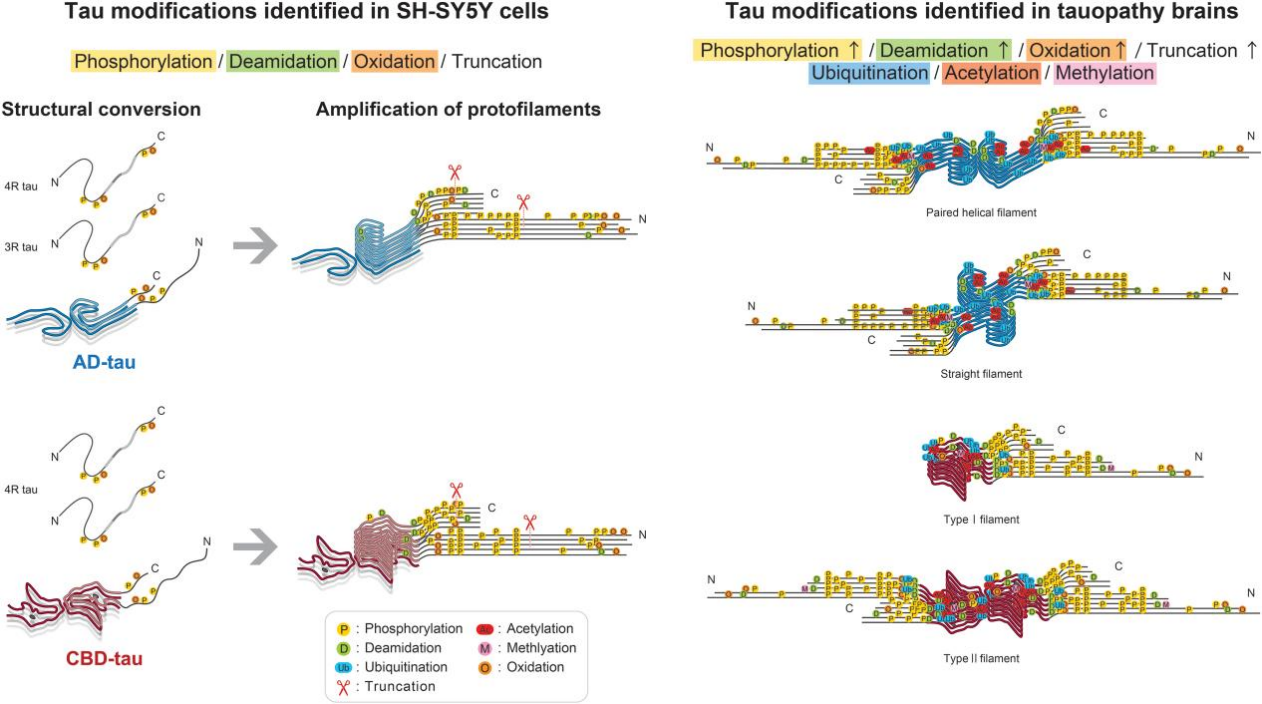
**Comparison of filamentous tau post-translational modifications in templated seeding in SH-SY5Y cells and in tauopathy brains.** When a template filament is present, the tau substrate matching the template is recruited and structurally converted in a template-dependent manner in SH-SY5Y cells. This leads to the formation of protofilaments stacked with identical filament cores. Phosphorylation and deamidation in the fuzzy coat of protofilaments increase with templated protofilament formation in cells. On the other hand, modifications at lysine residues, such as ubiquitination and acetylation, in addition to increased phosphorylation, deamidation, and oxidation in and around the filament core are detected in patient-derived tau filaments. Truncation of the N- and C-terminal tau in the fuzzy coat is also more advanced. Most post-translational modifications (PTMs) of the residues composing the filament core are located outside of the structured core, suggesting that distinct tau filament folds determine the PTM profile.

This incomplete PTM profile in seeded tau may reflect immaturity of the formed filaments in SH-SY5Y cells. Alzheimer’s disease and CBD filaments eventually appear in the patient’s brain as PHF/SF or type I (singlet)/type II (doublet), respectively.^34,36,44^ However, tau filaments seeded with Alzheimer’s disease and CBD filaments in this cellular model were identified only as protofilaments, suggesting that the interaction of two protofilaments requires some intracellular event(s), such as the N- and C-terminal truncation of tau. LC-MS/MS results show that the relative abundance of phosphorylation at the N- and C-terminal regions of seeded tau is higher than that of patient-derived tau (Fig. 3B). This is not thought to result from dephosphorylation caused by postmortem changes, since it has been reported that PHF-tau is hardly dephosphorylated.^45^ A possible explanation is that the sarkosyl-insoluble fraction extracted from the seeded cells contains a higher proportion of full-length tau than does that extracted from the patient’s brain. We have previously reported that insoluble tau seeded in SH-SY5Y cells exhibits a C-terminal fragment banding pattern similar to that of patient-derived tau, but it is possible that the fuzzy coat processing in SH-SY5Y cells may be not adequate for the two protofilaments to interact.^30^ Inadequate truncation of seeded full-length tau may also prevent the enzymatic PTMs in the filament core region. While tau I297-E391 peptides have been reported to form PHFs *in vitro*, tau I297-L441 peptides containing pseudo-phosphorylation at S396, S400, T403 and S404 form only protofilaments presenting the Alzheimer’s disease fold under the same *in vitro* conditions.^46,47^ These results support the idea that truncation of N- and C-terminal tau is involved not only in Alzheimer’s disease fold formation but also in PHF formation. Furthermore, the additional density surrounded by the side chains of three lysine residues (K290, K294 and K370) present in the CBD fold was not identical in seeded CBD-tau filaments.^36,37^ The hairpin or disordered structure of P364-G367 in seeded CBD filaments resulted in a smaller and less elongated additional density and incomplete structural inheritance of V363-T377.^37^ A possible role of lysine PTMs in the incorporation of an unknown molecule corresponding to this additional density is suggested by the detection of methylation at K294 and ubiquitination/acetylation at K370 in CBD-tau (Fig. 3A and Supplementary Fig. 6). To summarize our results, the presence of filamentous tau seeds in SH-SY5Y cells initially leads to the formation of tau protofilaments in a template-dependent manner, followed by increases in phosphorylation and deamidation in the fuzzy coat of the newly formed protofilaments (Fig. 4). Tau filaments extracted from tauopathy brains show a PTM pattern that reflects the filament core structure (Fig. 4). Although the factors involved in the maturation and doublet formation of tau filaments are unknown, possible PTM events occurring during these processes would include degradation of the fuzzy coat and lysine modifications. As it has been reported that the PTM profile changes depending on the Alzheimer’s disease Braak stage,^16^ further observations on tau filament formation using long-term-incubated cell culture models such as patient-derived induced pluripotent stem cells and animal models are required to reach a better understanding of the relationship between the maturation process of tau filaments and PTMs.

Our results do not imply that templated seeding is a consequence of PTMs, but PTMs may play a role in initial template (fold) formation that occurs in very early stages of pathogenesis. Pathological changes in the phosphorylation profile have been shown to alter the charge of soluble tau and potentially determine the direction of misfolding.^48,49^ Other PTMs may also modify the physical properties of soluble tau, which in turn may enhance the strain specificity.^50^ Acetylation at K281 is detected at a frequency of 100% in CBD-tau, whereas ubiquitination or acetylation at K280 is detected in FTDP-17-tau,^15^ indicating that PTMs at K280 or K281 may be responsible for the structural differences between the CBD and the AGD/FTDP-17/ARTAG folds (Supplementary Fig. 6).^12,36^ The prominence of ubiquitination or acetylation at K343, K353, K369, K370 and K375 also differed in each tauopathy (Fig. 3A and Supplementary Fig. 6). These disease-specific PTMs at the filament core region may not only structurally stabilize the fold itself, but also impact on the interaction between two protofilaments.^51^ It would be interesting to see what other factors, including the cell type and aging-related processes, affect (or modulate) the PTM profile.

Although the direct involvement of PTMs in complete structural inheritance in templated seeding remains an open question, it is also possible that PTMs affect the seeding efficiency. While both unmodified and modified tau seeds can cause seeded tau aggregation *in vitro* and *in vivo*, PTMs have been reported to exert inhibitory or promoting effects.^30,31,52,53^ Synthetic tau filaments with phosphorylation at 1–3 residues among S258, S262 and S356 exhibited lower seeding activity than unphosphorylated filaments *in vitro* and in HEK-293T biosensor cells, and the inhibitory effect on seeding activity varied depending on the phosphorylation sites.^54^ Phosphorylated residues close to the filament core region, such as S258 and S262, may have a more proximate effect on the conformational change of substrate tau.^54,55^ It has also been reported that dephosphorylated AD-tau markedly reduces propagation efficiency in the mouse brain.^56^ Acetylated tau seeds have been shown to promote seeded aggregation of full-length tau in HEK-QBI-293 and -293A cell models.^57,58^ Thus, the possible contribution of PTM to the seeding efficiency should be further investigated.

In conclusion, many tau PTMs in common with those of the introduced patient-derived tau seeds are reproduced in the seeding model of SH-SY5Y cells, and PTMs that characterize the disease appear to be mimicked as the maturation of tau filaments progresses. Our findings in this cellular model suggest that templated amplification of tau filaments is the underlying process leading to the formation of tau pathologies composed of structurally and biochemically identical tau filaments. Therefore, the design of aggregation inhibitors and antibody drugs based on tau filament core structure could be a promising approach to suppress templated tau seeding in the patient’s brain.

## Supplementary Material

awad272_Supplementary_Data

## Data Availability

All raw data used for figure generation in this study can be obtained by contacting the corresponding author.
