# Supplementary material for: Distinct tau folds initiate templated seeding and alter the post-translational modification profile
Source: Brain. 2023 Aug 10;146(12):4988–99. doi: 10.1093/brain/awad272 (PMC10690015; doi:10.1093/brain/awad272)
Supplement: awad272_Supplementary_Data [file awad272_supplementary_data.zip › brain-2023-00910-File007.pdf]

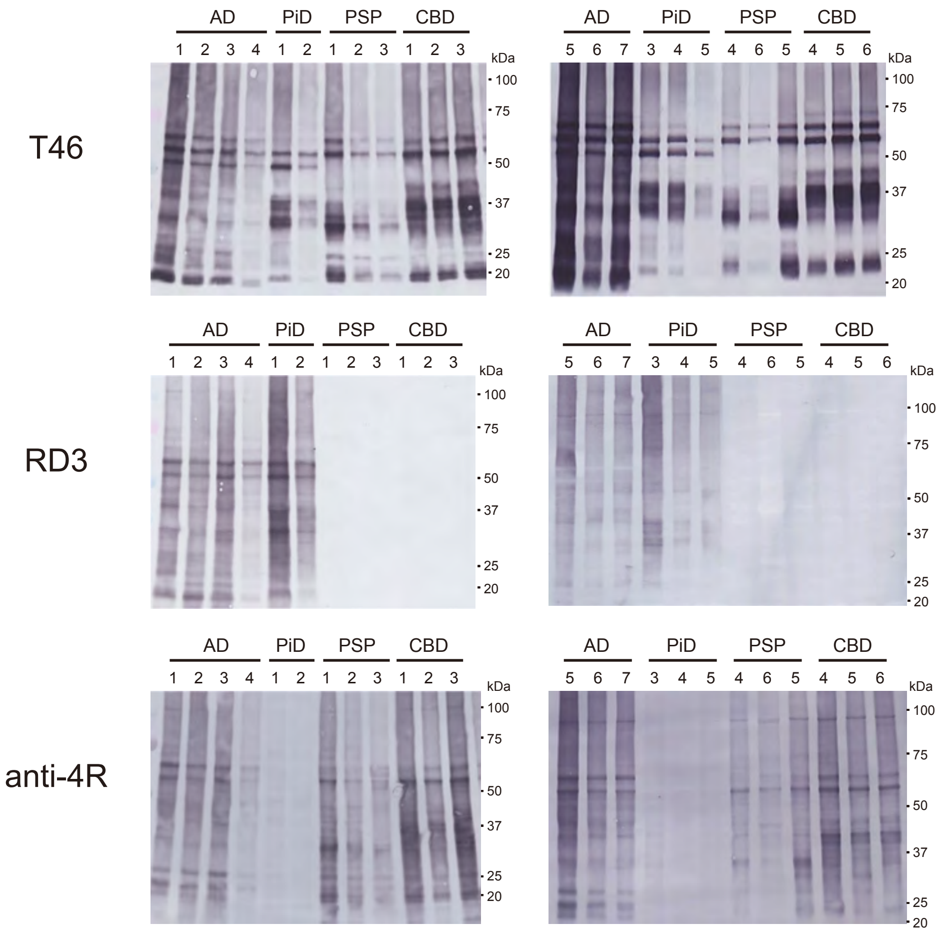

Supplemental Figure 1 Biochemical characterization of sarkosyl-insoluble tau extracted from tauopathy brains

Sarkosyl-insoluble fractions prepared from patients' brains used in this study were analyzed by immunoblotting with T46, RD3 and anti-4R antibodies.

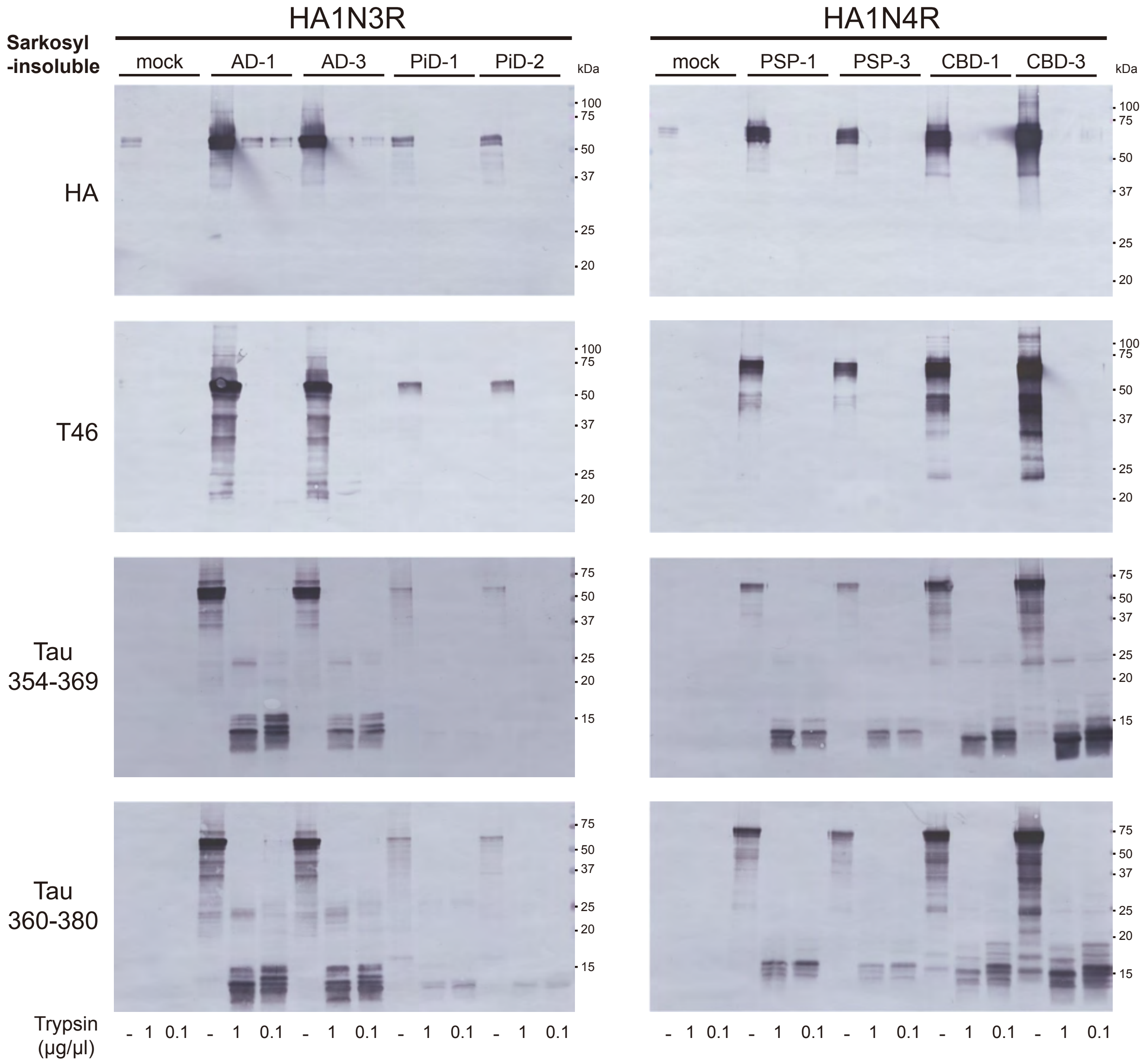

Supplemental Figure 2 Trypsin-resistant banding patterns of insoluble tau extracted from SH-SY5Y cells seeded with patient-derived tau strains

Sarkosyl-insoluble fractions extracted from SH-SY5Y cells seeded with patient-derived tau seeds were treated with 0.1 or 1 μg/μl trypsin. Immunoblot analysis of untreated and trypsin-treated samples. N-Terminal tau and C-terminal tau were detected using anti-HA and T46 antibodies, respectively. Trypsin-resistant tau bands were detected with Tau 354-369 and Tau 360-380 antibodies.

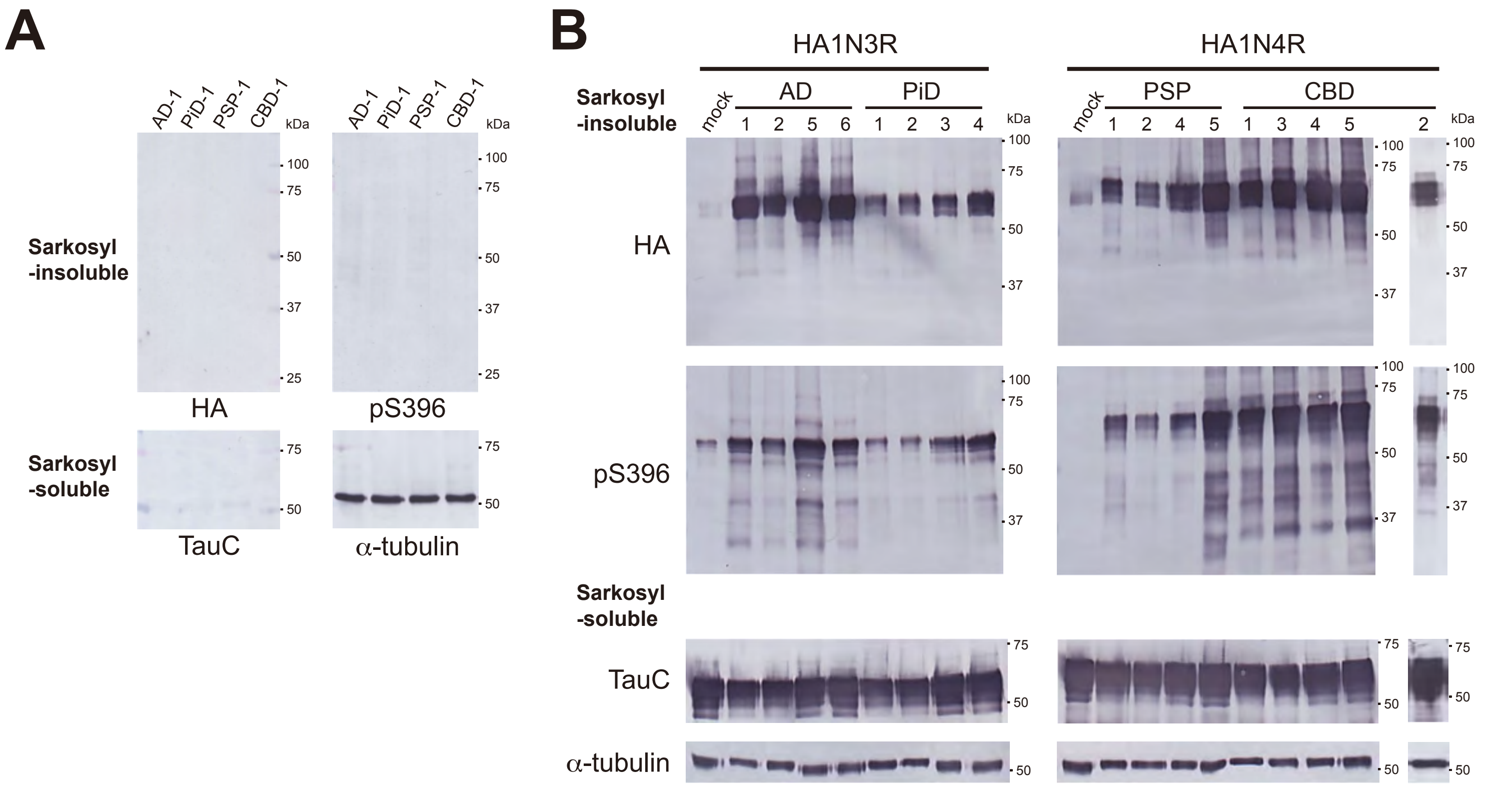

Supplemental Figure 3 Immunoblot analysis of insoluble tau extracted from SH-SY5Y cells and used for LC/MS-MS analysis

A Sarkosyl-insoluble fractions extracted from patients' brains were introduced into SH-SY5Y cells without transient expression of tau. Immunoblot analysis of sarkosyl-insoluble fractions and sarkosyl-soluble fractions extracted from cells seeded with patient-derived tau seeds. Insoluble tau was detected with anti-HA and pS396 antibodies. Total tau was detected with TauC antibody.

B Sarkosyl-insoluble fraction extracted from patients' brains was introduced into SH-SY5Y cells transiently expressing HA1N3R or HA1N4R. Immunoblot analysis of sarkosyl-insoluble fractions and sarkosyl-soluble fractions extracted from mock cells and cells seeded with patient-derived tau seeds after incubation for 3 days. Insoluble tau was detected with anti-HA and pS396 antibodies. Total tau was detected with TauC antibody.

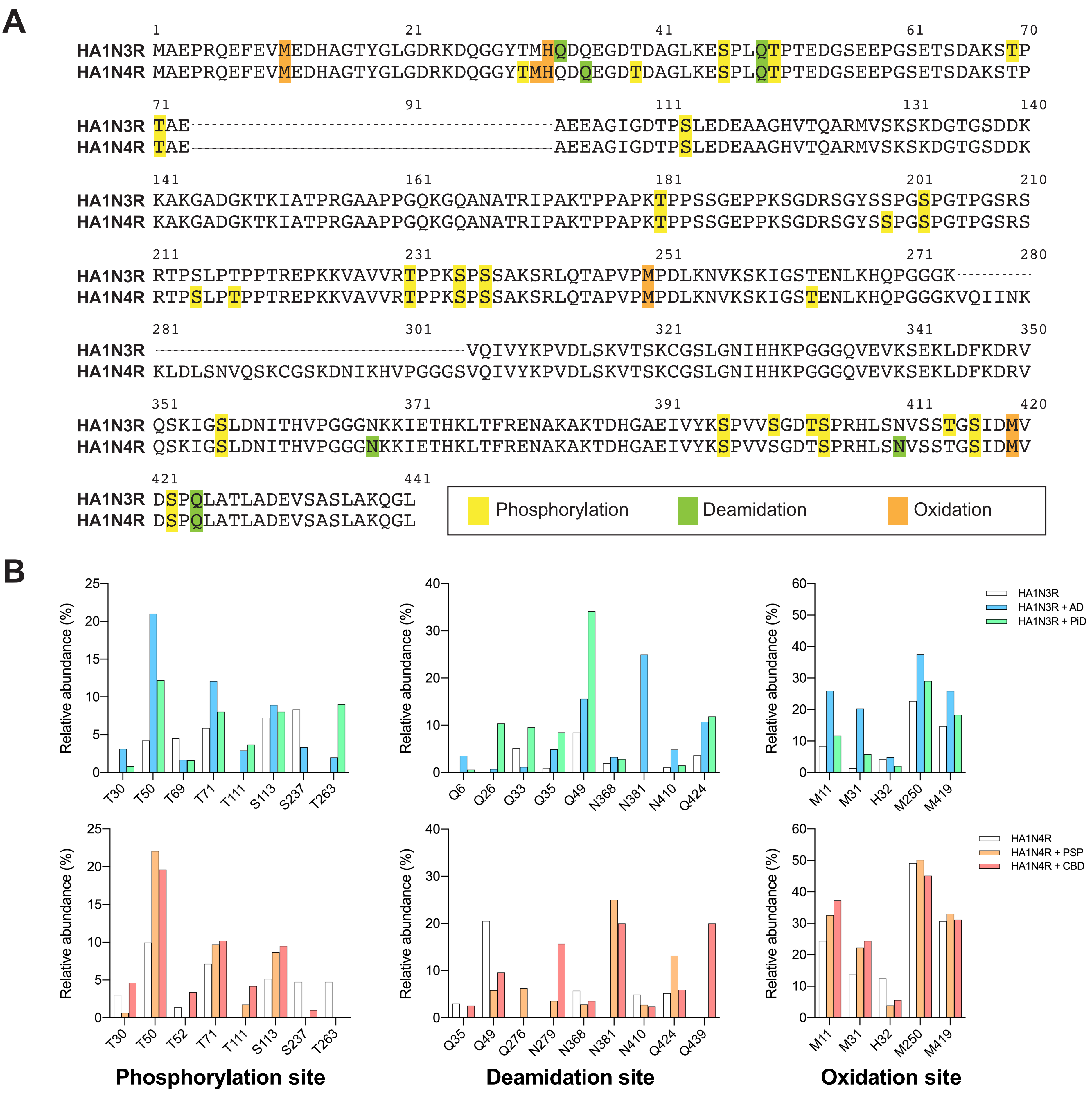

Supplemental Figure 4 Tau PTMs of insoluble tau extracted from SH-SY5Y cells expressing full-length tau

A Sequence alignments of 1N3R in sarkosyl-insoluble tau extracted from SH-SY5Y cells expressing HA1N3R (HA1N3R) and 1N4R in sarkosyl-insoluble tau extracted from SH-SY5Y cells expressing HA1N4R (HA1N4R). PTMs detected by LC-MS/MS analysis at >3% relative abundance are shown. Phosphorylation, deamidation and oxidation sites are highlighted in yellow, bright green and orange, respectively.

B The relative abundances of tau phosphorylation, deamidation and oxidation sites detected in the sarkosyl-insoluble fractions extracted from SH-SY5Y cells expressing HA1N3R (HA1N3R) without seeds and seeded with AD-tau (HA1N3R + AD) and PiD-tau (HA1N3R + PiD) (upper) and from SH-SY5Y cells expressing HA1N4R without seeds (HA1N4R) and seeded with PSP-tau (HA1N4R + PSP) and CBD-tau (HA1N4R + CBD) (lower). Relative abundance (%) was calculated from the ratio of modified peptides / (modified + unmodified peptides). The results are expressed as means (*n* = 3-4).

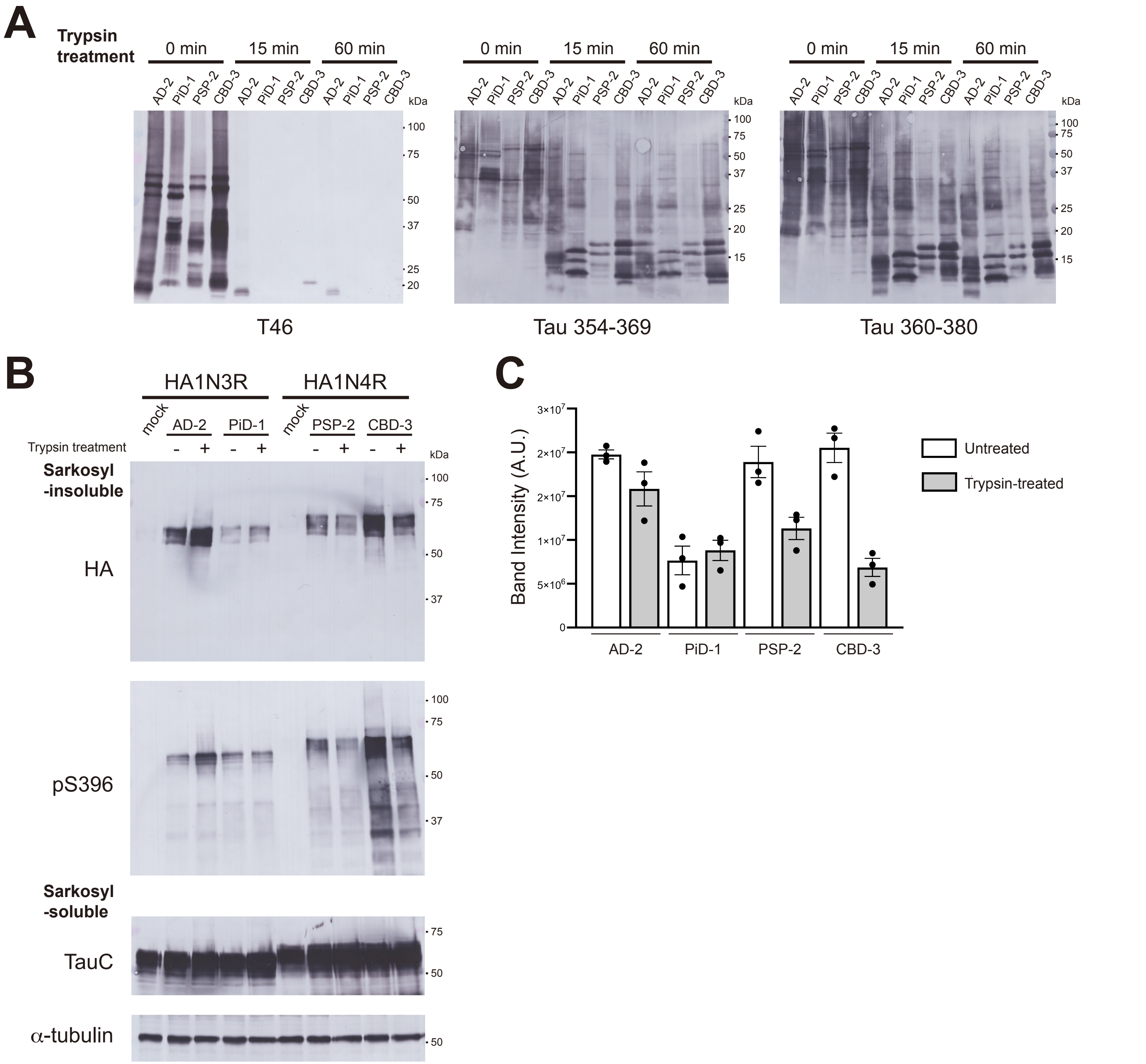

Supplemental Figure 5 Seeded tau aggregation induced by trypsin-treated patient-derived tau strains in SH-SY5Y cells

A Immunoblot analysis of the sarkosyl-insoluble fractions extracted from patients' brains before and 15 and 60 minutes after trypsin treatment. Full-length tau and C-terminal tau fragments were detected with T46 antibody. Trypsin-resistant tau bands were detected with tau 354-369 and tau 360-380 antibodies.

B Untreated and trypsin-treated patient-derived tau seeds were introduced into SH-SY5Y cells transiently expressing HA3R1N or HA4R1N. Immunoblot analyses of sarkosyl-insoluble fractions and sarkosyl-soluble fractions extracted from mock cells, and cells seeded with untreated or trypsin-treated tau seeds. Insoluble tau was detected with anti-HA and pS396 antibodies. Total tau was detected with TauC antibody.

C The band intensities of the immunoblots with anti-HA antibody shown in B were quantified. The results are expressed as means ± SEM (*n* = 3).

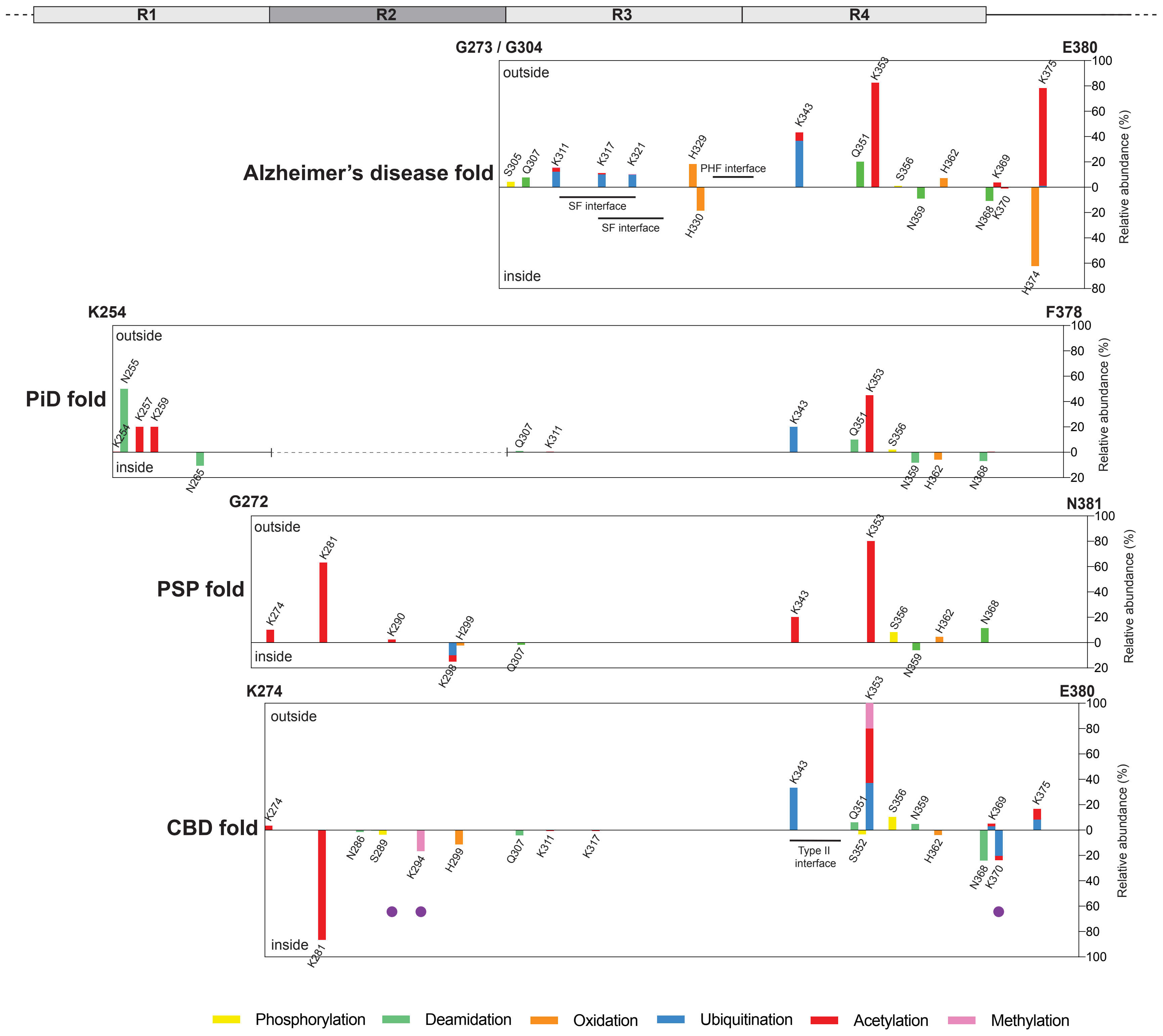

Supplemental Figure 6 Tau PTMs in the core structure of tau filaments extracted from tauopathy brains
The relative abundances of tau phosphorylation, deamidation, oxidation, ubiquitination, acetylation and methylation sites detected in the filament core region of insoluble tau extracted from tauopathy brains are shown. Ascending and descending bars indicate PTM residues located outside or inside the structured filament core, respectively. PHF or SF interfaces in the Alzheimer's disease fold and Type Ⅱ interface in the CBD fold indicate the regions of the inter-protofilament interface. The purple dot in the CBD fold represents lysine residues with side chains surrounding the additional density. Relative abundance (%) was calculated from the ratio of modified peptides / (modified + unmodified peptides). The results are expressed as means (*n* = 5-6).

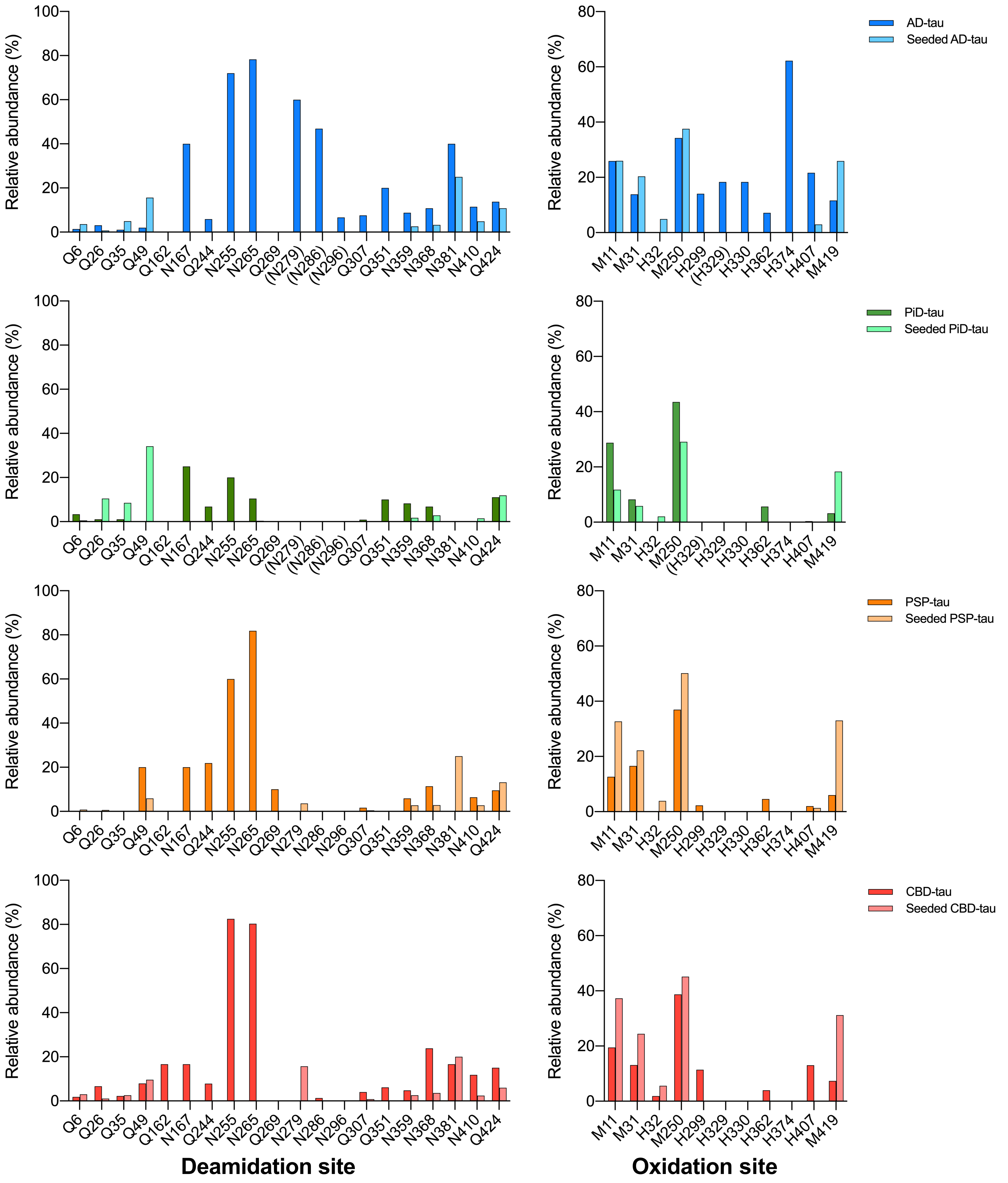

Supplemental Figure 7 Deamidation and oxidation frequencies detected in templated seeding in SH-SY5Y cells and in tauopathy brains

Comparison of deamidation and oxidation frequencies detected in sarkosyl-insoluble tau derived from tauopathy brains and from SH-SY5Y cells seeded with patient-derived tau (seeded tau). Relative abundance (%) was calculated from the ratio of modified peptides / (modified + unmodified peptides). The results are expressed as means (n = 4-6). Bracketed residues indicate residues not included in the seeded tau.

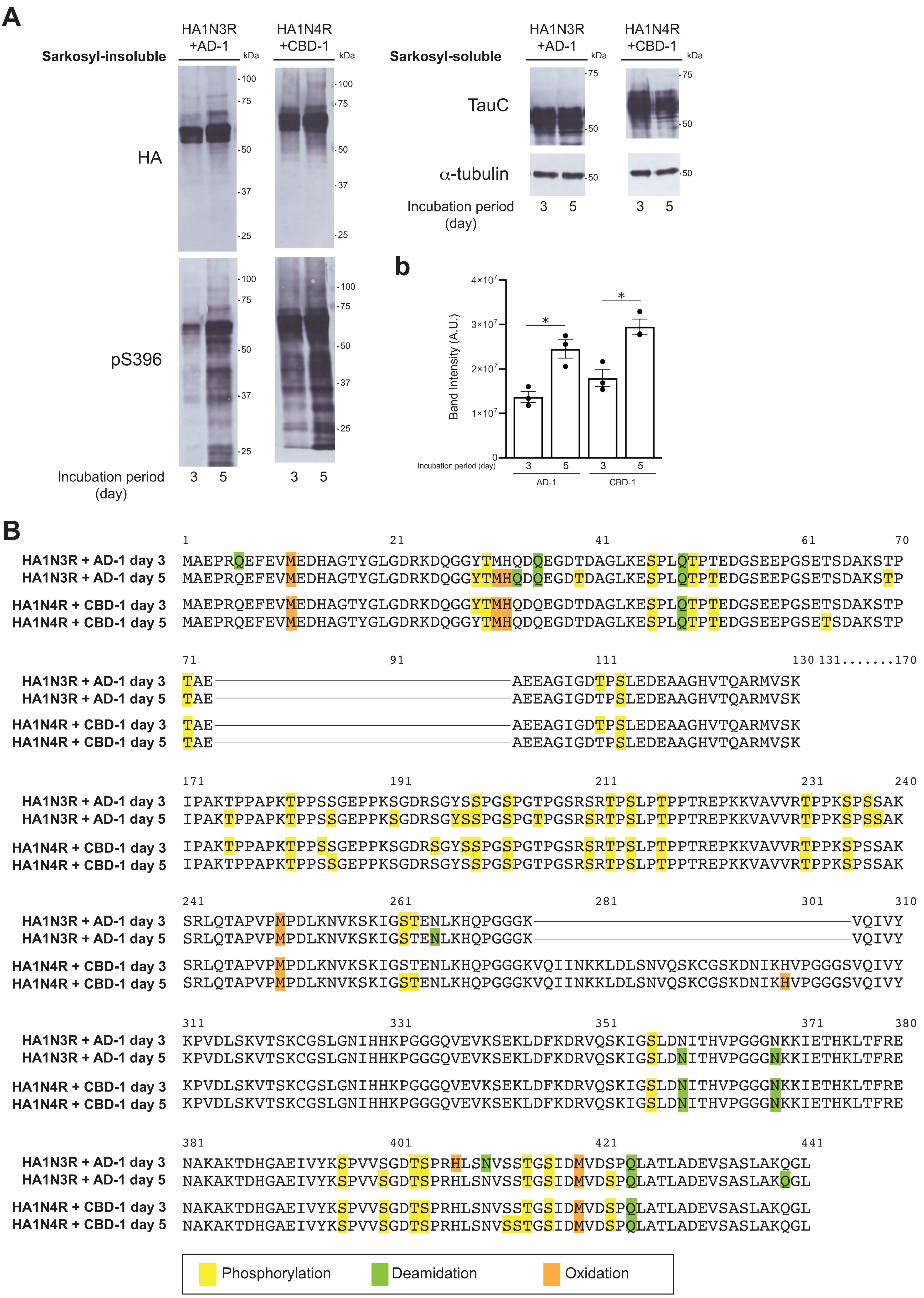

Supplemental Figure 8 Time-dependent alteration of tau PTMs of insoluble tau extracted from SH-SY5Y cells seeded with patient-derived tau strains

A Sarkosyl-insoluble fraction extracted from AD-1 or CBD-1 was introduced into SH-SY5Y cells transiently expressing HA1N3R or HA1N4R. Immunoblot analysis of sarkosyl-insoluble fractions and sarkosyl-soluble fractions extracted from transfected cells after incubation for 3 and 5 days. Insoluble tau was detected with anti-HA and pS396 antibodies. Total tau was detected with TauC antibody.

B The band intensities of the immunoblots with anti-HA antibody shown in A were quantified. The results are expressed as means ± SEM (n = 3). Statistical analysis was performed with an unpaired t-test (*$P < 0.01$)

C Sequence alignments of 1N3R in sarkosyl-insoluble tau extracted from SH-SY5Y cells expressing HA1N3R seeded with AD-1 (HA1N3R + AD-1 day 3 and HA1N3R + AD-1 day 5) and 1N4R in sarkosyl-insoluble tau extracted from SH-SY5Y cells expressing HA1N4R seeded with CBD-1 (HA1N4R + CBD-1 day 3 and HA1N4R + CBD-1 day 5). PTMs detected by LC-MS/MS analysis at >3% relative abundance are shown. Phosphorylation, deamidation and oxidation sites are highlighted in yellow, bright green and orange, respectively. No PTMs were detected in residues 131 to 170.

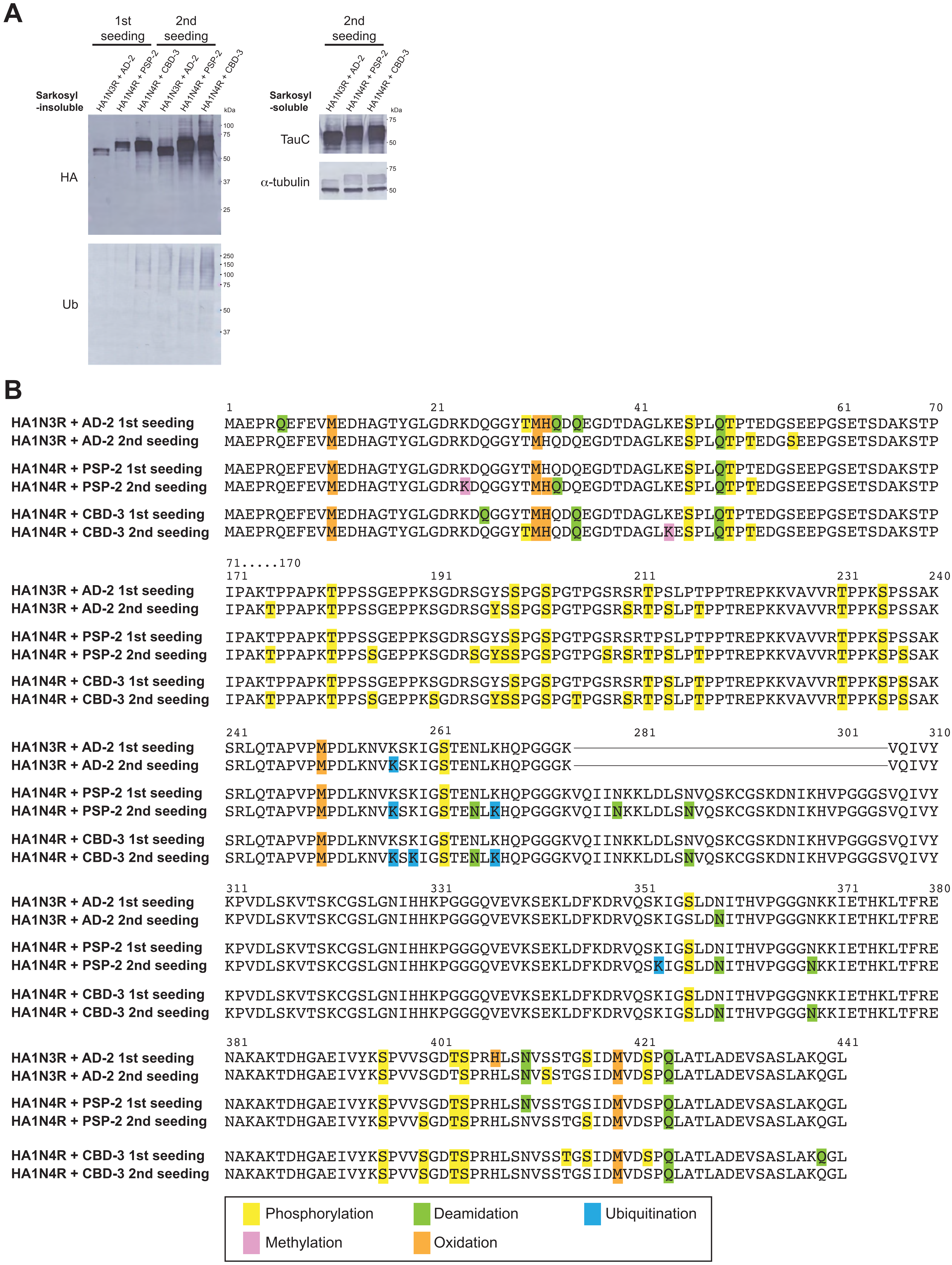

Supplemental Figure 9 Comparison of tau PTMs identified in initial and secondary seeded aggregates in SH-SY5Y cells

A Sarkosyl-insoluble fractions extracted from cells transfected with patient-derived tau seeds (1st seeding) were introduced into SH-SY5Y cells transiently expressing HA1N3R or HA1N4R (2nd seeding). Immunoblot analysis of sarkosyl-insoluble and sarkosyl-soluble fractions extracted from cells obtained at 3 days after the 1st seeding and 2nd seeding. Insoluble tau was detected with anti-HA antibody. Ubiquitinated proteins were detected with anti-ubiquitin (Ub) antibody. Total tau was detected with TauC antibody.

B Sequence alignments of 1N3R in sarkosyl-insoluble tau extracted from SH-SY5Y cells expressing HA1N3R seeded with AD-2 (1N3R + AD-2 1st seeding and 1N3R + AD-2 2nd seeding) and 1N4R in sarkosyl-insoluble tau extracted from SH-SY5Y cells expressing HA1N4R seeded with PSP-2 (1N4R + PSP-2 1st seeding and 1N4R + PSP-2 2nd seeding) and CBD-3 (1N4R + CBD-3 1st seeding and 1N4R + CBD-3 2nd seeding). PTMs detected by LC-MS/MS at >3% relative abundance are shown. Phosphorylation, deamidation, ubiquitination, methylation and oxidation sites are highlighted in yellow, bright green, blue, pink and orange, respectively.
