# Supplementary material for: Distinct tau folds initiate templated seeding and alter the post-translational modification profile
Source: Brain. 2023 Aug 10;146(12):4988–99. doi: 10.1093/brain/awad272 (PMC10690015; doi:10.1093/brain/awad272)
Supplement: awad272_Supplementary_Data [file awad272_supplementary_data.zip › brain-2023-00910-File009.pdf]

Source images of immunoblots

Fig. 1B

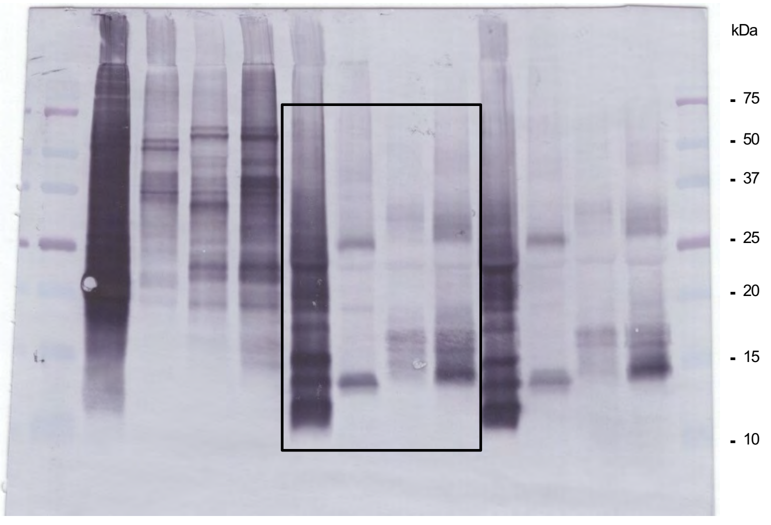

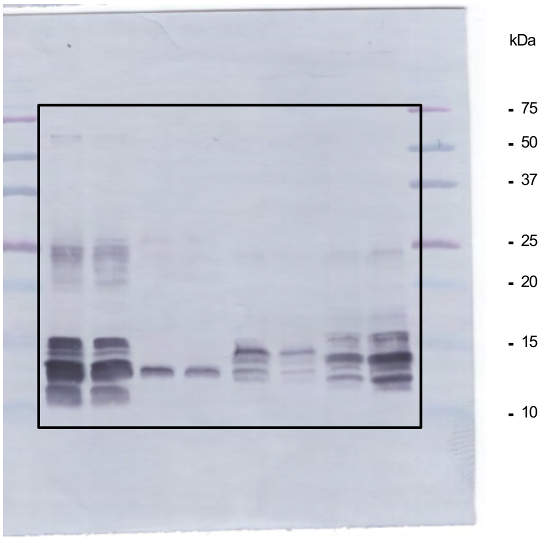

Source images of immunoblots

Supplemental Fig. 1

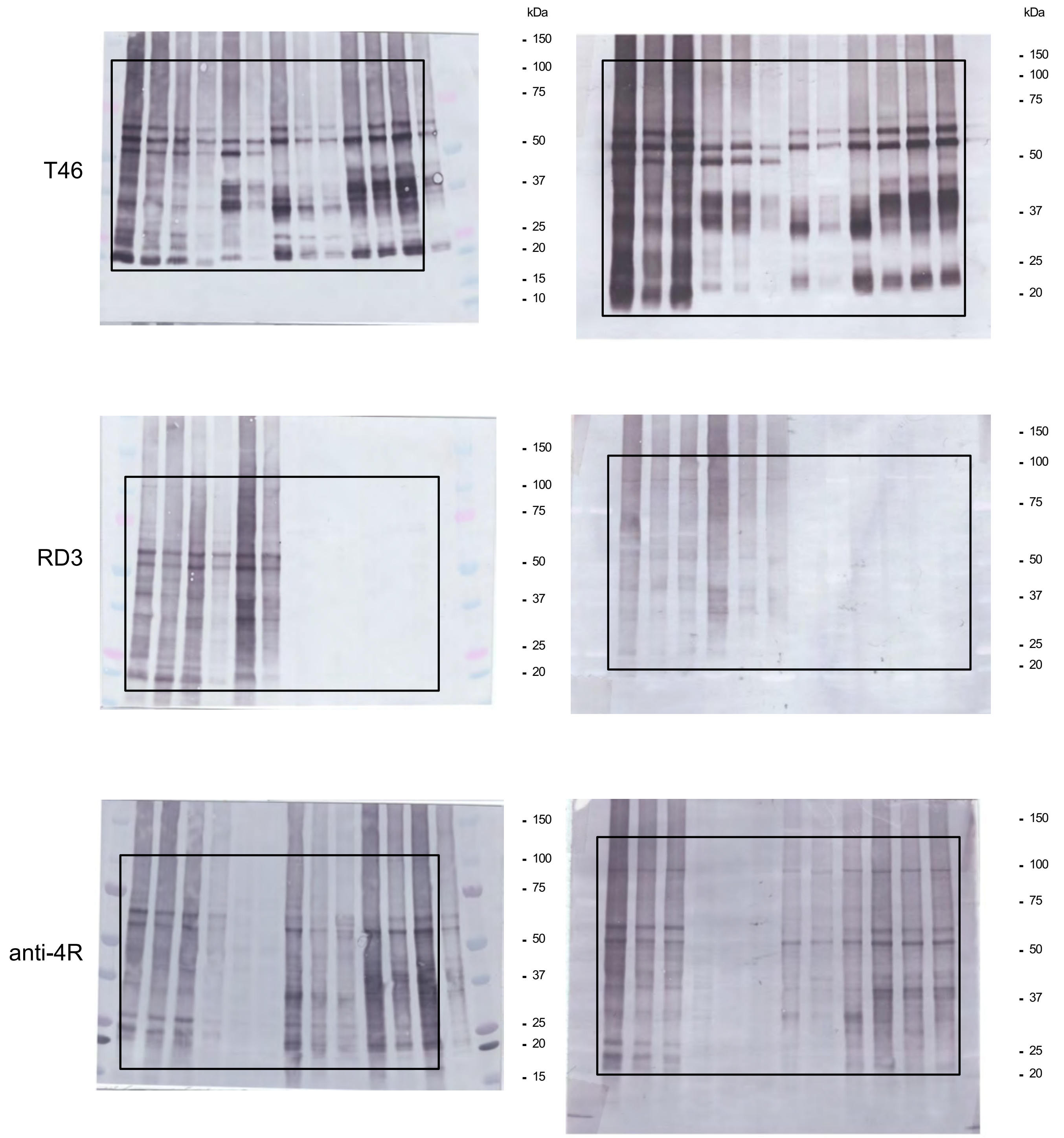

Source images of immunoblots

Supplemental Fig. 2

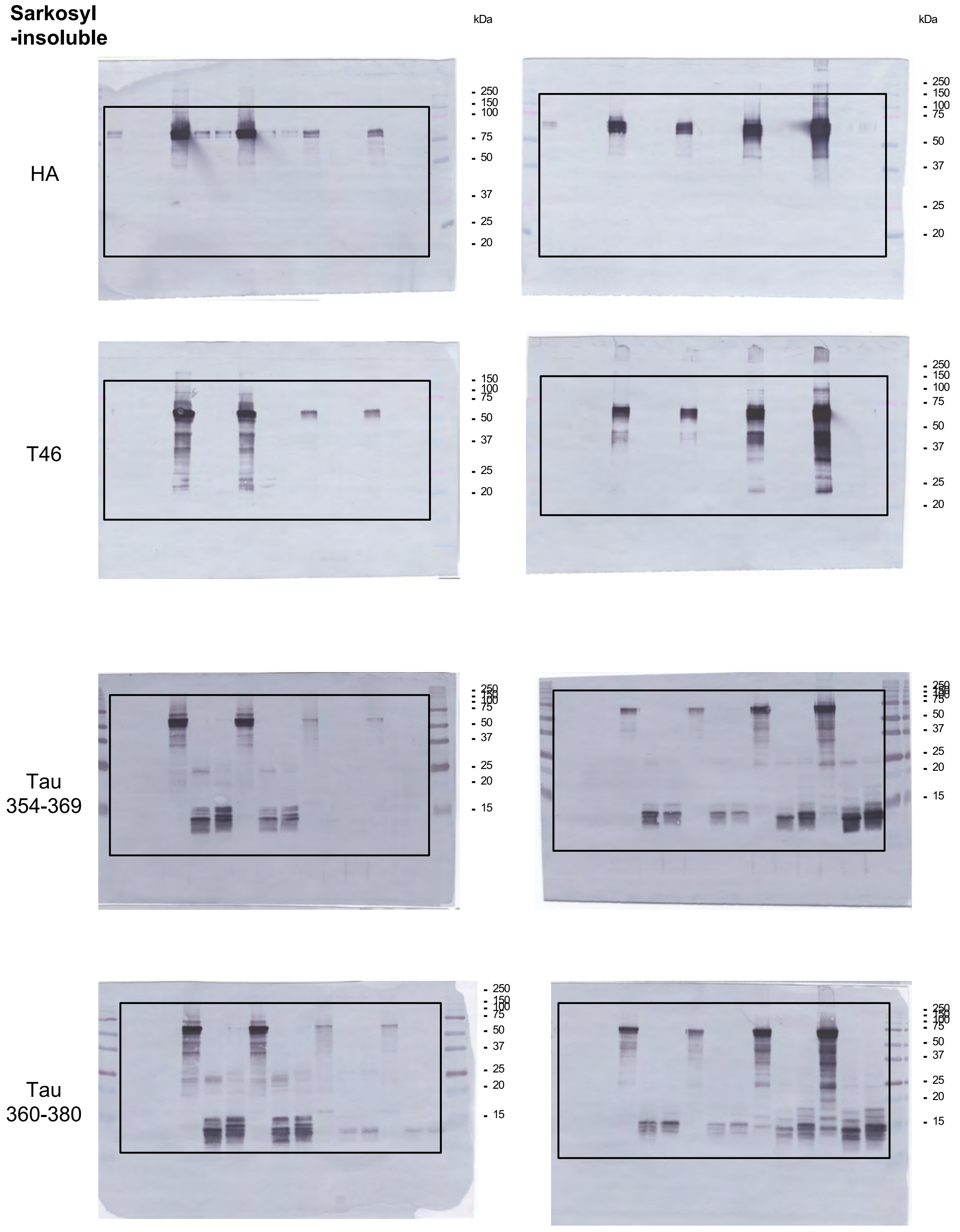

Source images of immunoblots

Supplemental Fig. 3A

Sarkosyl -insoluble

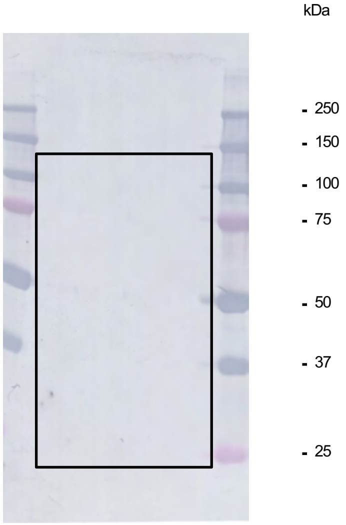

HA

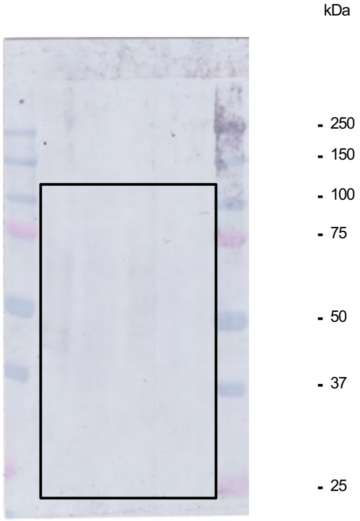

pS396

Sarkosyl -soluble

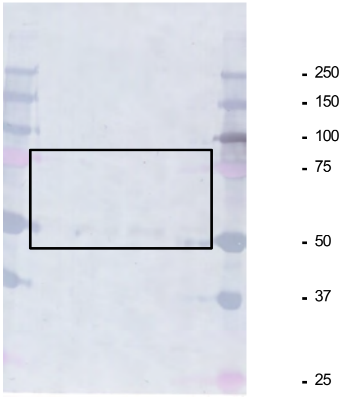

TauC

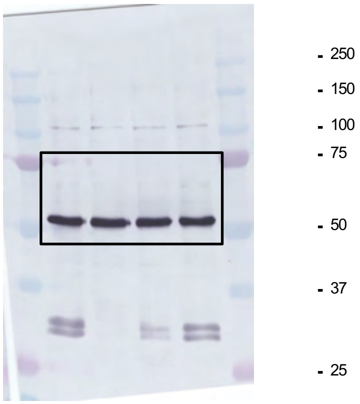

α-tubulin

Source images of immunoblots

Supplemental Fig. 3B

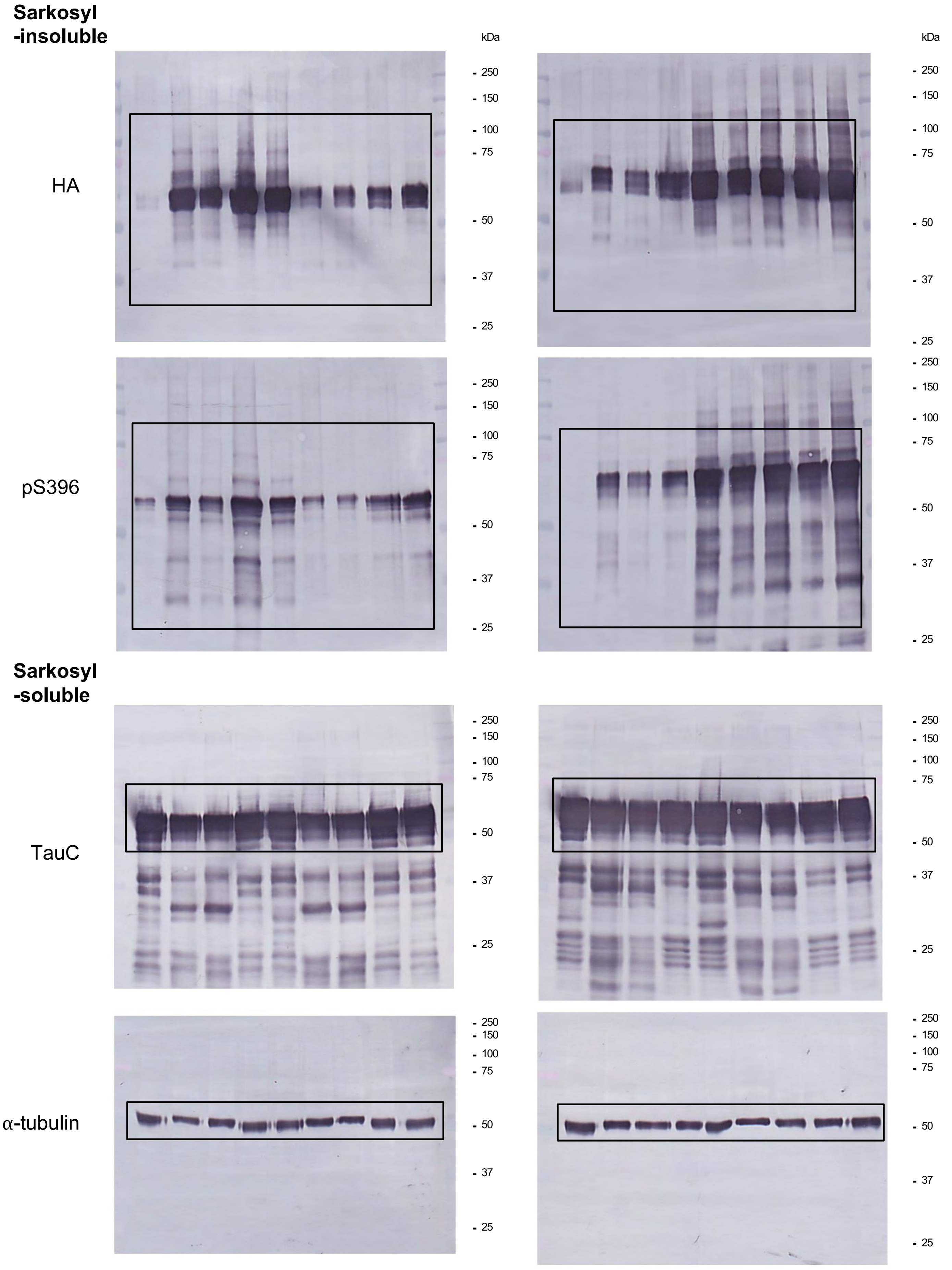

Source images of immunoblots

Supplemental Fig. 3B

Sarkosyl -insoluble

CBD

kDa

2

HA

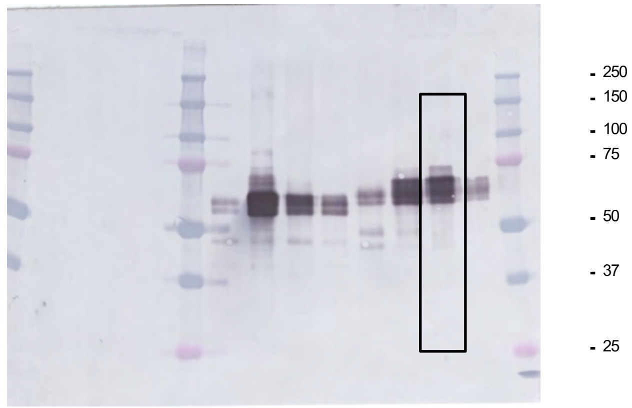

pS396

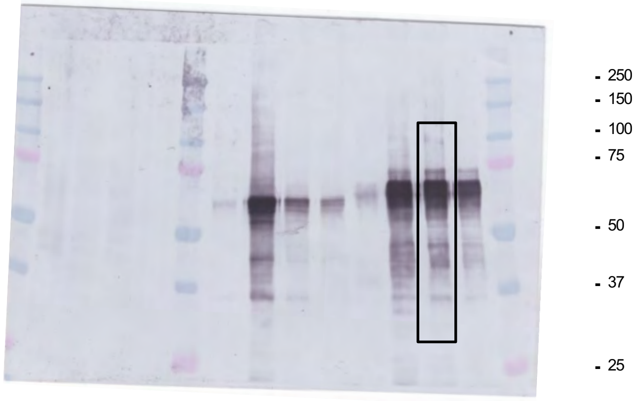

Sarkosyl -soluble

TauC

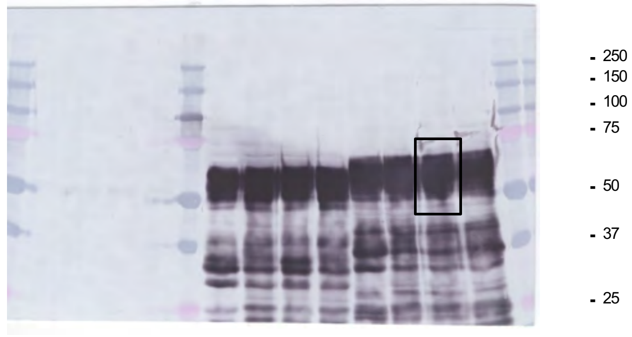

α-tubulin

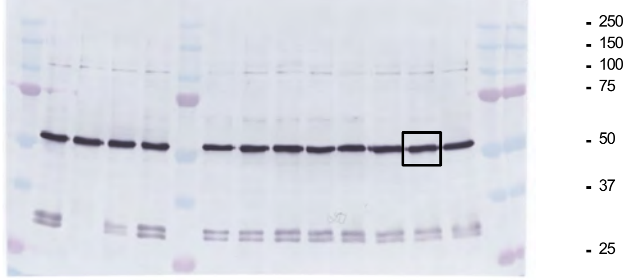

Source images of immunoblots

Supplemental Fig. 5A

T46

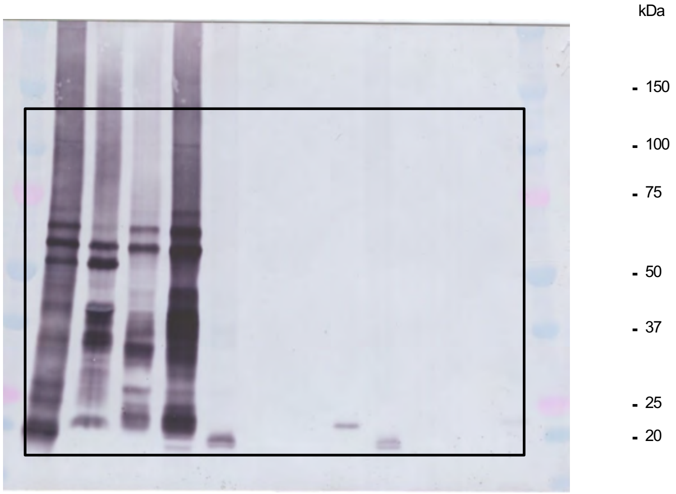

Tau
354-369

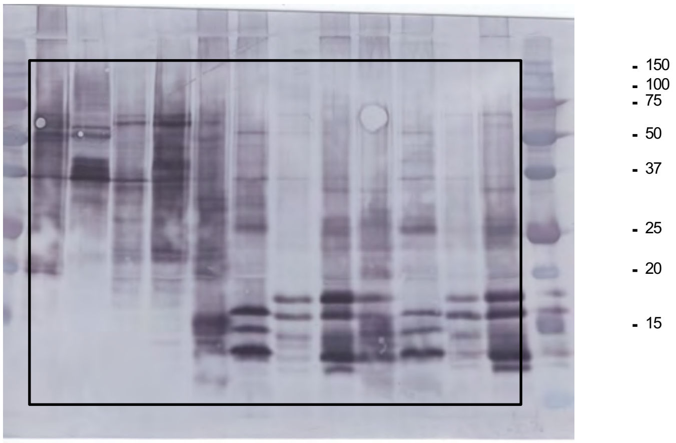

Tau
360-380

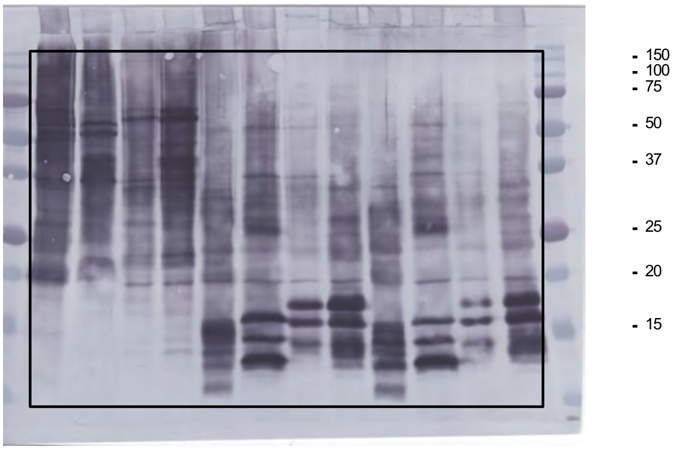

Source images of immunoblots

Supplemental Fig. 5B

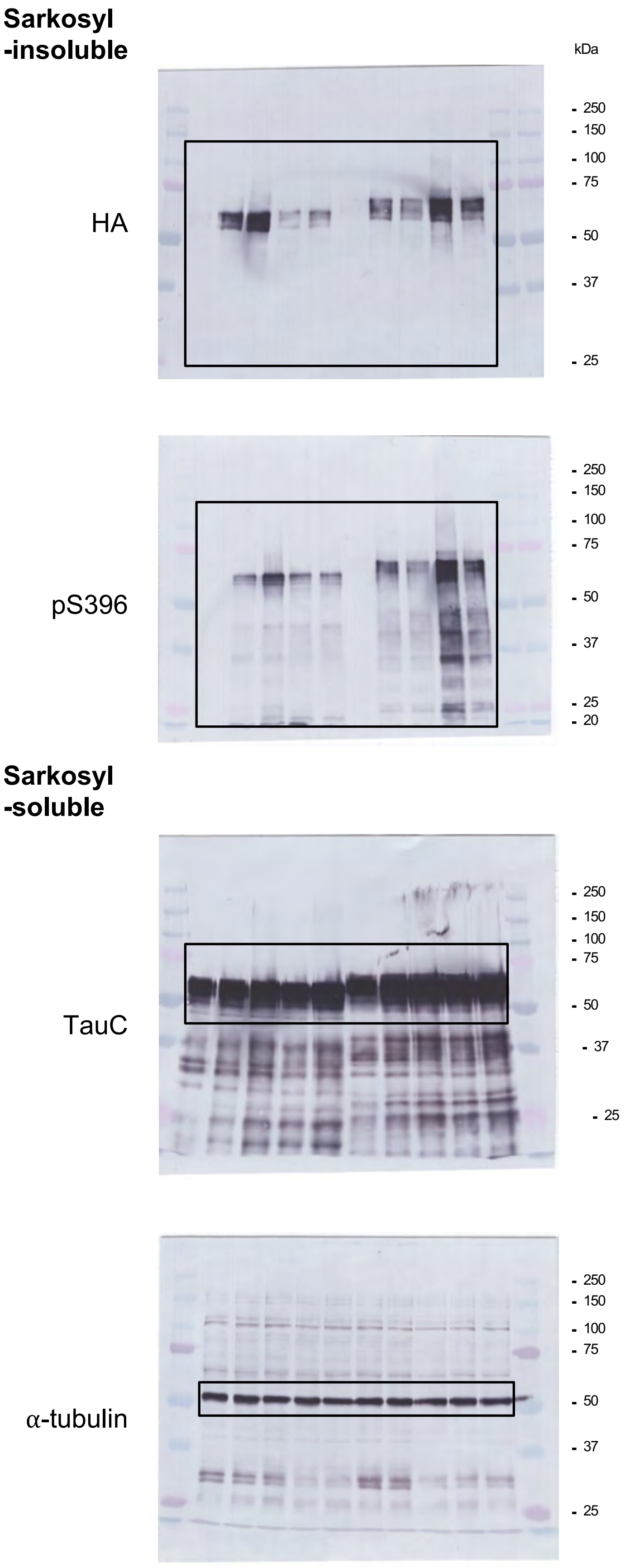

Source images of immunoblots

Supplemental Fig. 8A

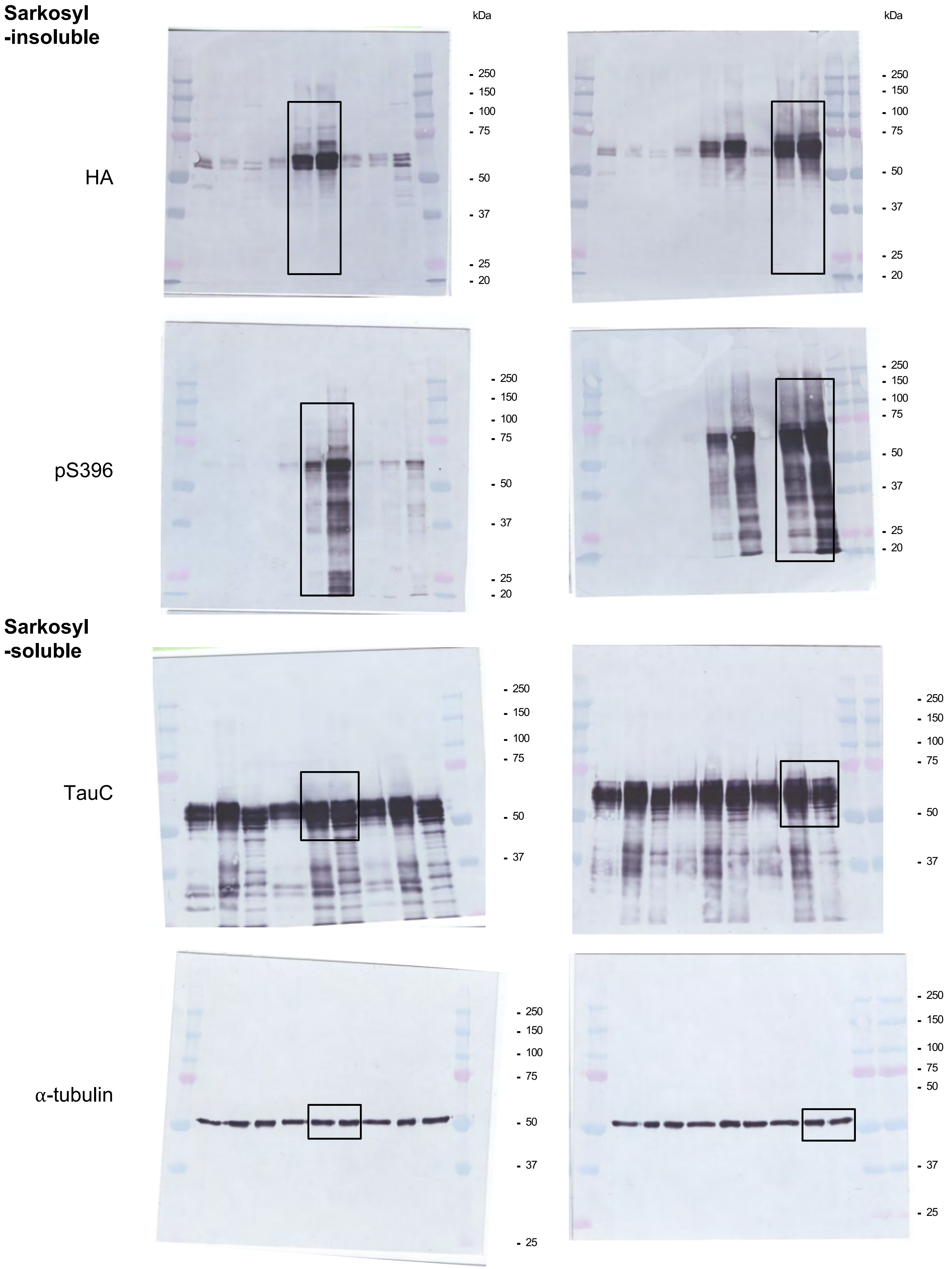

Source images of immunoblots

Supplemental Fig. 9A

Sarkosyl-insoluble

HA

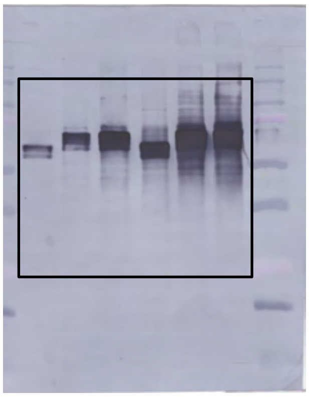

kDa
- 250
- 150
- 100
- 75
- 50
- 37
- 25

Ub

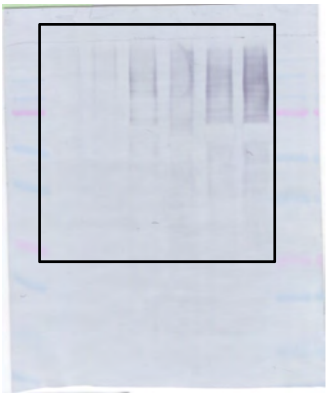

- 250
- 150
- 100
- 75
- 50
- 37
- 25

Sarkosyl-soluble

TauC

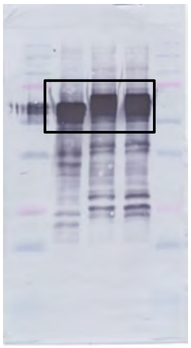

- 250
- 150
- 100
- 75
- 50
- 37
- 25
- 20

α-tubulin

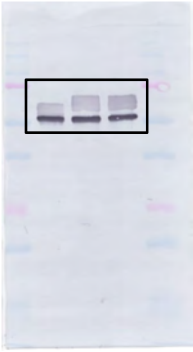

- 250
- 150
- 100
- 75
- 50
- 37
- 25
